# Stacking correlation length in single-stranded DNA

**DOI:** 10.1093/nar/gkae934

**Published:** 2024-10-26

**Authors:** Xavier Viader-Godoy, Maria Manosas, Felix Ritort

**Affiliations:** Small Biosystems Lab, Departament de Física de la Matèria Condensada, Facultat de Física, Universitat de Barcelona, Carrer de Martí i Franquès, 1, 08028 Barcelona, Spain; Dipartimento di Fisica e Astronomia Galileo Galilei, Università degli Studi di Padova, Via Francesco Marzolo, 8, 35131 Padova, Italy; Small Biosystems Lab, Departament de Física de la Matèria Condensada, Facultat de Física, Universitat de Barcelona, Carrer de Martí i Franquès, 1, 08028 Barcelona, Spain; Institut de Nanociència i Nanotecnologia, Universitat de Barcelona, 08029 Barcelona, Spain; Small Biosystems Lab, Departament de Física de la Matèria Condensada, Facultat de Física, Universitat de Barcelona, Carrer de Martí i Franquès, 1, 08028 Barcelona, Spain; Institut de Nanociència i Nanotecnologia, Universitat de Barcelona, 08029 Barcelona, Spain

## Abstract

Base stacking is crucial in nucleic acid stabilization, from DNA duplex hybridization to single-stranded DNA (ssDNA) protein binding. While stacking energies are tiny in ssDNA, they are inextricably mixed with hydrogen bonding in DNA base pairing, making their measurement challenging. We conduct unzipping experiments with optical tweezers of short poly-purine (dA and alternating dG and dA) sequences of 20–40 bases. We introduce a helix-coil model of the stacking–unstacking transition that includes finite length effects and reproduces the force-extension curves. Fitting the model to the experimental data, we derive the stacking energy per base, finding the salt-independent value $\Delta G_0^{ST}=0.14(3)$ kcal/mol for poly-dA and $\Delta G_0^{ST}=0.07(3)$ kcal/mol for poly-dGdA. Stacking in these polymeric sequences is predominantly cooperative with a correlation length of ∼4 bases at zero force . The correlation length reaches a maximum of ∼10 and 5 bases at the stacking–unstacking transition force of ∼10 and 20 pN for poly-dA and poly-dGdA, respectively. The salt dependencies of the cooperativity parameter in ssDNA and the energy of DNA hybridization are in agreement, suggesting that double-helix stability is primarily due to stacking. Analysis of poly-rA and poly-rC RNA sequences shows a larger stacking stability but a lower stacking correlation length of ∼2 bases.

## Graphical Abstract

**Graphical Abstract:**
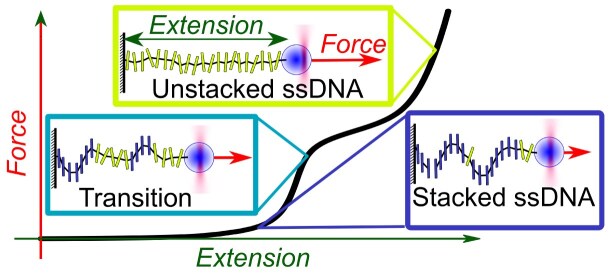

## Introduction

Nucleic acids (NAs) participate in information transfer and regulatory genomic processes that require the readout of the bases. Molecular reactions in NAs involve opening double-stranded (ds) helical structures, converting them into single-stranded forms (ssDNA and ssRNA), making bases accessible to the cell machinery (1). Stacking forces are crucial in the hybridization reaction where the two complementary strands form a duplex also stabilized by hydrogen bonding (2,3). Base stacking is also essential for understanding allostery and other molecular actions at a distance. In DNA, stacking regulates protein binding during replication and recombination (4). Stacking of the nascent RNA chain modulates co-transcriptional RNA folding, whereas for mRNA, it impacts co-translational protein folding (5,6).

Besides, stacking in ssDNA promotes the formation of structures (7–9). Poly-deoxyadenine (poly-dA) sequences form single and double-stranded helices (10) stabilized by stacking interactions, similarly to poly-adenine (poly-rA) sequences (11). In contrast, poly-deoxycytosine (poly-dC) and poly-deoxyguanine (poly-dG) sequences form complex structures such as i-tetraplexes and G-quadruplexes, relevant for the regulation of gene expression (12–14). These polynucleotide structures emerge from the interplay between stacking and non-canonical base pairing. The structural diversity of ssDNA is also relevant for many applications such as DNA origami (15), DNA nano switches (16), synthetic molecular motors (17) and immunodetection (13). Despite their importance, direct measurements of stacking energies in ssDNA remain scarce.

Base pairs in dsDNA form adjacent stacks that stabilize the double helix. Stacking energies in dsDNA have been measured using DNA origami nanotubes manipulated with optical tweezers (18), and indirectly through melting experiments (19) and by mechanically unzipping single DNA molecules (20,21). The energies of the ten different combinations of stacks in the nearest-neighbor (NN) model have been determined, finding values in the range of 1−3 kcal/mol (22,23). Various studies indicate that stacking is the main contribution to the free energy of hybridization (7,8,24–26). However, these measurements do not permit us to infer the much lower stacking energies of ssDNA, approximately ∼0.1 kcal/mol (1). Stacking of ssDNA has been measured with several techniques: calorimetry (27), nuclear magnetic resonance (28), X-ray diffraction (29), single-molecule fluorescence (30), atomic force microscopy (31) and magnetic tweezers (32). Measurements of force-extension curves (FECs) with magnetic tweezers on purine-rich sequences (32) show a cooperative stacking-unstacking (S-U) transition around 20 pN. Most NA studies have focused on homopolymeric sequences containing purines or pyrimidines. Poly-dA shows the largest level of stacking (31), whereas poly-uracil (poly-U), poly-deoxythymidine (poly-dT) and mixed poly-pyrimidine (poly-pyr) sequences do not show stacking (30–32). On the other hand, studies with short DNA oligonucleotides of mixed sequences demonstrate that the minimal purine-rich motif for stacking must contain at least four bases (27).

Here, we investigate stacking in ssDNA by measuring the FECs of short poly-purine sequences of varying lengths using optical tweezers. The FECs exhibit a shoulder at a given force, where the convexity of the FEC changes, a feature of the stacking–unstacking transition. We introduce a helix-coil model for stacking that reproduces the experimental FECs of poly-dA and poly-dGdA and the observed finite length effects. The model contains two energy parameters: the stacking energy per base ε_*ST*_ and the cooperativity of stacking between neighboring bases γ_*ST*_. The sensitivity of the force data and the model’s features permit us to accurately derive the stacking free energies and correlation length at different salt conditions. Notably, we find a maximum in the correlation length at a force value directly related to the energy parameters of the model, ε_*ST*_ and γ_*ST*_. Finally, we further validate the model by analyzing previous results on different ssDNA (32) and ssRNA (33) sequences. Our results show that γ_*ST*_ is systematically larger than ε_*ST*_, indicating that stacking cooperativity is the primary source of stabilization in NAs.

## Materials and methods

### Experimental setup and sample preparation

Experiments were performed using the miniTweezers setup (34), which consists on two counter-propagating laser beams (P = 200 mW, λ = 845 nm) that follow a symmetrical optical path and are focused on creating a single optical trap in a microfluidics chamber (35). The molecular construct was tethered between polystyrene beads coated with Streptavidin (2.0 μm *Kisker Biotech*) or anti-Digoxigenin (3.0 μm *Kisker Biotech*), keeping the former fixed via air suction to a micropipette. The latter was trapped by the optical trap whose exerted force and position were measured by using Position Sensitive Detectors (36). The instrument has a 0.1 pN and 1 nm resolution at a 1 kHz acquisition rate. Beads were attached to the DNA construct by labelling each molecular end with Biotin or Digoxigenin. The DNA hairpins used in this work were synthesized by following a similar procedure to what was done in Ref. (37), annealing three oligonucleotides (Supplementary Sec. S1). The first and longer one contains the hairpin region and the flanking 29b of the handles (with a Biotin in its 5’ end, *Merck Sigma-Aldrich*), and a tail of digoxigenins is added via Terminal Transferase enzyme (*Merck Sigma-Aldrich*). The second oligo is a short 14b segment complementary to the second handle, while the third oligo is fully complementary to the first handle and to the 15b of the second one, leaving a 4b spacer between them. The experiments were all performed at 25^○^C. Those with varying NaCl concentrations (10, 50, 100, 500 and 1000 mM) contained also (10 mM Tris-HCl pH7.5, EDTA 1 mM, 0.01% sodium azide), while the experiments with MgCl_2_ were performed at 10 mM Tris-HCl pH7.5, 0.01%NaN_3_ (sodium azide) and 10 mM MgCl_2_.

### Extension determination

**Figure 1.**
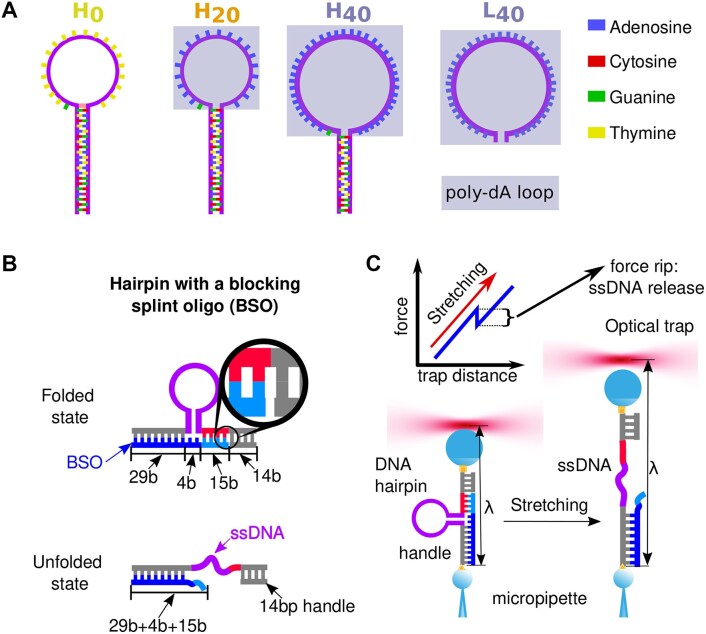
**Hairpin sequences, molecular construct and experimental setup**. (**A**) Schematic depictions of the four hairpins studied. Gray boxes show the poly-dA regions of the sequence. Hairpins are named by the number of purines in the loop: *H*_0_ (20bp stem and 20b dT-loop); *H*_20_ (20bp stem and 20b purine-loop -1dG, 19dA-); *H*_40_ (15bp stem and 40b purine-loop -1dG, 39dA-). *L*_40_ consists of a 40dA loop without stem. **(B**) Scheme of the folded (top) and unfolded (bottom) states of the hairpins with the 48b BSO. **C**. Sketch of the optical tweezers setup. Left: a DNA hairpin is attached to two beads using specific linkages, one is held by a micropipette and the other by the optical trap. Right: the trap distance λ is moved away from the micropipette, while the applied force on the DNA hairpin increases, until it unfolds, and a force rip is observed in the FDC (top left).

**Figure 2.**
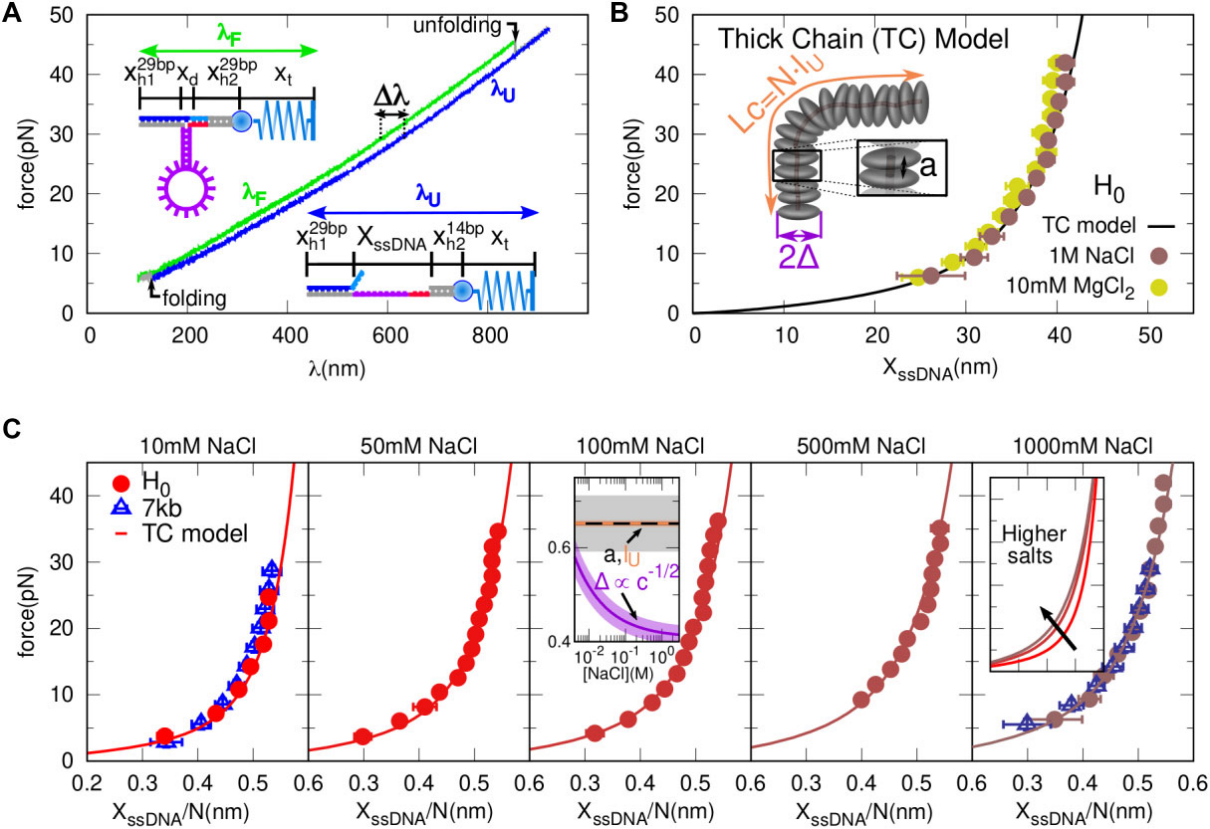
**Unstacked ssDNA elasticity**. (**A**) A typical FDC for *H*_0_. In light green (dark blue blue) are shown the unfolding (folding) branches, with λ_*F*_ (λ_*U*_) being the trap position (trap-pipette distance). The elastic contributions to the trap position λ at the F(U) branches are schematically depicted on top (bottom). Arrows indicate the jump in force when the molecule unfolds or folds, changing from one branch to another. These forces dictate the limits for applicability of the two branches method. (**B**) FEC of *H*_0_ for 10 mM MgCl_2_ (light yellow dots) and 1M NaCl (dark brown dots). Black lines show the fit of the TC model to the NaCl data. The inset shows a schematic depiction of the Thick Chain (TC) model, with its three parameters: the disk radius Δ, the spacing *a*, and the total contour length, *L*_*c*_. (**C**) FECs per base *x*_ssDNA_/*N*, for *H*_0_ (red filled circles) and 7kbp hairpin [(blue empty triangles, Ref. (9)] for different NaCl concentration, with their fits to the TC model (continuous lines). The inset of the central panel shows the salt dependence of the fitting TC model parameters. Shadowed areas are the statistical errors obtained by bootstrapping (*N* = 500). The right inset shows how the theoretical FECs change with salt concentration. The error bars are the standard errors of the molecules studied at each condition (Supplementary Figure S3, Supplementary Table S6).

To obtain the ssDNA extension of the unfolded hairpin, we employ the two-branches method (38), which is based on analyzing the hairpin’s pulling curves. Pulling experiments, where the force is cyclically increased and decreased to unzip and re-zip the hairpin, were carried out using the blocking splint oligo (BSO) method (39) to increase the hairpin unfolding forces. Briefly, a 48-base oligonucleotide is hybridized to the ssDNA handles at the two flanking sides of the hairpin (Figure 1B): 29b hybridize to the left handle (gray-blue duplex in Figure 1B, top); 15b hybridize to the right handle (cyan-red duplex in Figure 1B, top); a spacer of 4b connects both sides to accommodate the diameter of the DNA hairpin stem. When the force increases above ∼40 pN, the shorter 15b right handle unbinds and the hairpin unfolds keeping the longer 29b left handle hybridized (Figure 1C, right). Upon releasing the force, the hairpin refolds, and the 15b right oligo hybridizes again (cyan segment in Figure 1C, left). The higher stability of dsDNA to shearing increases the hairpin unfolding force from ∼15 to ∼40pN, allowing us to obtain precise ssDNA FECs for short molecules (See Supplementary Section S7) in a wide range of forces (5 ≤ *f* ≤ 45 pN). The hairpin force-distance curve (FDC) is divided into two branches: the folded (F), where the hairpin is folded, and the unfolded (U), where the hairpin is unfolded, Figure 2A. The molecular extension of the ssDNA at a given force, *X*_ssDNA_(*f*), can be obtained by subtracting the relative trap position (λ) of the U (λ_*U*_ ) and F (λ_*F*_) branches. As shown in Figure 2A, the optical trap position at the F branch is given by $\lambda _F=x_{h1}^{\rm 29bp}(f)+x_d(f)+x_{h2}^{\rm 29bp}(f)+x_t(f)$, with $x_{h1(h2)}^{\rm 29bp}$, *x*_*d*_ and *x*_*t*_ being the extension of dsDNA handles 1(2), the oriented hairpin diameter and the bead position in the trap, respectively. Whereas the optical trap position at the U branch is given by $\lambda _U=x_{h1}^{\rm 29bp}(f)+X_{\rm ssDNA}(f)+x_{h2}^{\rm 14bp}(f)+x_t(f)$. Therefore,


(1) \begin{eqnarray*}
X_{\rm ssDNA}(f)=\lambda _U(f)-\lambda _F(f)+x_{\rm d}(f)+x_{\rm dsDNA}^{\rm 15bp}(f),
\end{eqnarray*}

where the oriented hairpin diameter *x*_d_(*f*) is modeled as a freely-jointed chain with a single monomer of 2nm length (40); whereas $x_{\rm dsDNA}^{\rm 15bp}(f)$ is the extension of the 15bp segment of dsDNA (Supplementary Section S2).

**Figure 3.**
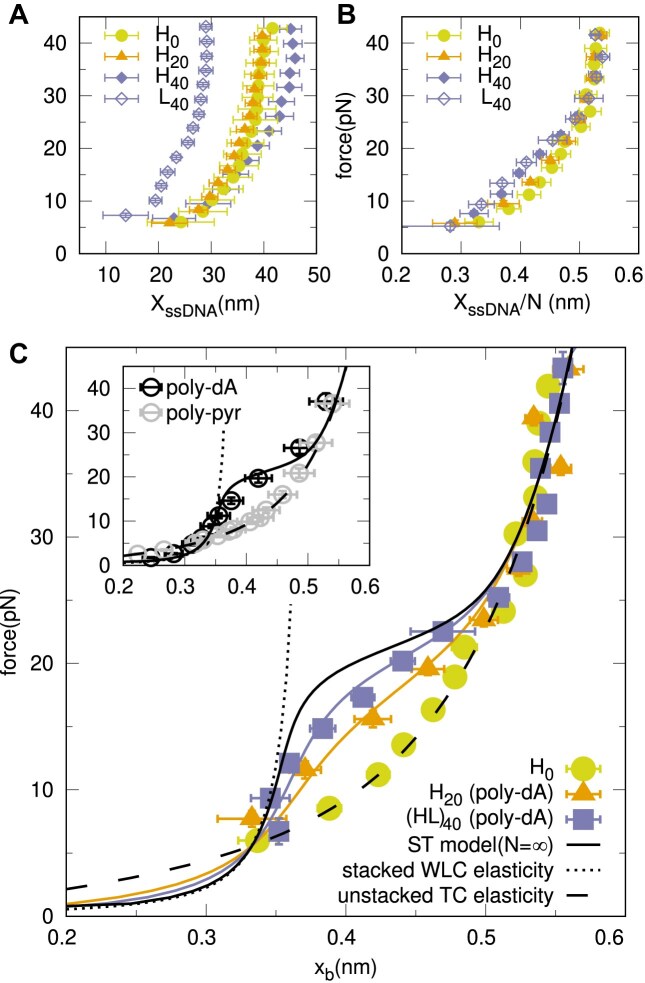
**poly-dA stacking** (**A**) FECs of *H*_0_ (yellow circles), *H*_20_ (orange triangles), *H*_40_ (blue filled rhombi) and *L*_40_ (blue empty rhombi). (**B**) Re-scaled FECs of the molecules shown in panel A. (**C**) Re-scaled FECs for varying lengths: *H*_0_ (re-scaled over its total number of bases) and *H*_20_ and an average of *H*_40_ and *L*_40_ (re-scaled extensions of their poly-dA loops) with their respective fits. Color code as in b. Inset shows the comparison of the theoretical re-scaled FECs for the unstacked state (dashed) and the predicted for the infinite ST-model, compared with magnetic tweezers data (32) for a polypyrimidine and poly-dA ssDNA sequences.

To compare the elastic behavior of the different ssDNA loop sequences and lengths, we use the re-scaled extension per base in the loop *x*_*b*_, as shown in Figures 3C, 5A and 6A for poly-dA and poly-dGdA loops. It is computed by subtracting the elastic contribution of the bases not belonging to the loop (the hairpin stem + 15b of the right handle) to the measured extension *X*_ssDNA_:


(2) \begin{eqnarray*}
x_b=\frac{X_{\rm ssDNA}-\left(N-N_{\rm loop}\right)x_U}{N_{\rm loop}},
\end{eqnarray*}

**Figure 4.**
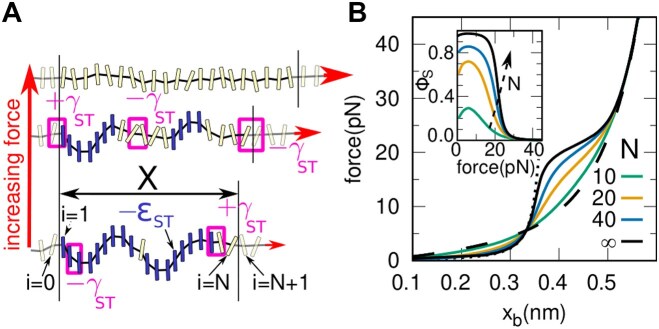
**Stacking (ST) model**. (**A**) Schematic depiction of the model for *N* = 20 bases. The bases are in either a stacked (dark blue) or unstacked (light yellow) state. The former are favored energetically by ε_ST_, while adjacent bases are energetically favored (penalized) with γ_ST_ if they do (not) share state. As force increases, the longer unstacked state is energetically favored. (**B**) Theoretical FECs for varying lengths (color lines). Dashed and dotted lines represent the completely unstacked and unstacked elasticity, respectively. The black continuous line shows the model prediction for a domain of *N* → ∞. The inset shows the fraction of bases in the stacked state, ϕ_*S*_, as a function of the force (same color code as the main panel).

**Figure 5.**
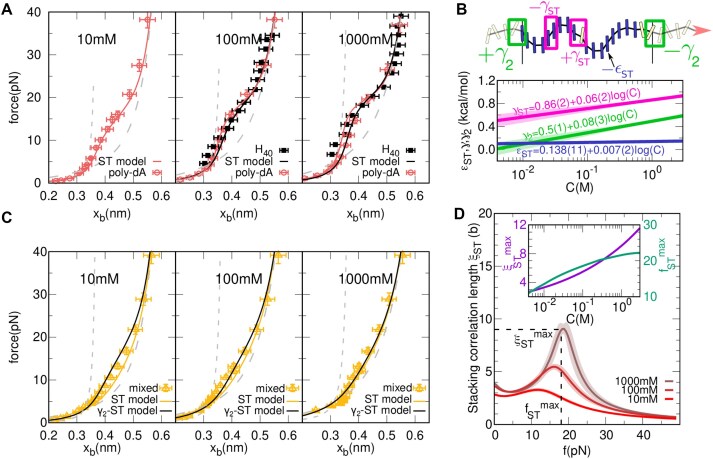
**Salt dependence of base stacking**. (**A**) FECs of the poly-dA loop for the *H*_40_ (black squares) and poly-dA from Ref. (32) (empty red circles) for 10, 100 and 1000mM NaCl concentration. The gray dashed and dotted lines represent the elasticity of the unstacked and stacked state using the TC and WLC models, respectively. Solid curves are the fitting ones using the finite (black) and infinite (red) model (50mM and 500mM curves for *H*_40_ shown in Supplementary Figure S8). (**B**) (top) Schematic depiction of the cooperativity between adjacent domains for the ST-γ_2_ model. (bottom) Salt dependence of the stacking energy per base, ε_ST_ and interaction energies between purines (γ_ST_) and the purine-pyrimidine boundary one (γ_2_). (**C**) Results for the few kb mixed sequence of Ref. (32) containing the repetitive 28 b motif (**AAGAG**TAT**GGAAAG**T **AAAAGAAA**T**AAAG**) with three poly-purine regions of 9,6 and 8 b (bold letters) for 10, 100 and 1000 mM NaCl (orange triangles). Solid orange and black lines correspond to the fits of the finite ST-model and the ST-γ_2_ models, respectively. The gray dashed and dotted lines represent the elasticity of the unstacked and stacked state using the TC and WLC models, respectively. **D** Correlation lengthfrom the ST model, Eq. (5), as a function of the force for 10, 100 and 1000 mM NaCl concentration. Inset: Theoretical predictions for the infinite model of the maximum stacking correlation length ($\xi ^{max}_{\textrm {ST}}$, magenta) and the force at which it peaks ($f^{\textrm { }max}_{\textrm { }ST}$) as a function of the salt concentration, *C*. Statistical uncertainties are shown as shadowed areas.

**Figure 6.**
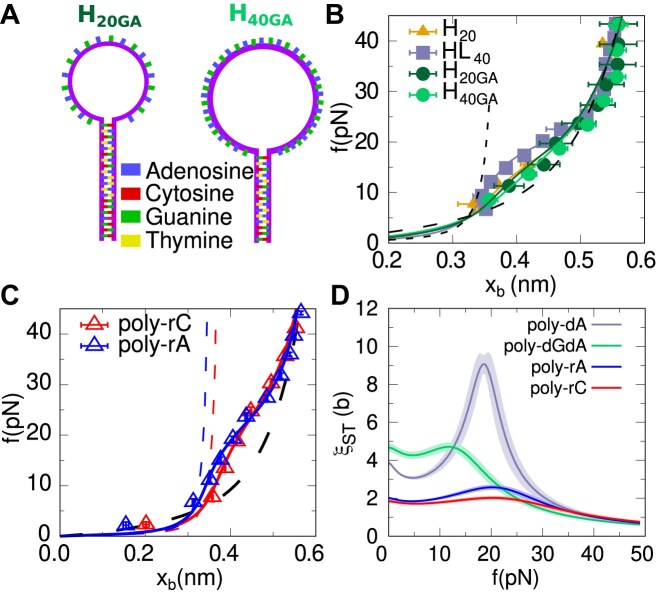
**Stacking in ssRNA compared to ssDNA**. (**A**) Schematic depictions of the two hairpins with poly-dGdA motifs in the loop. (**B**) Re-scaled FECs per base for the dGdA loops of *H*_20GA_ (dark green), *H*_40GA_ (light green). As a comparison, data for the 20b and 40b dA loops (orange triangles and gray squares) is shown. Dashed lines represent the elasticity of the stacked and unstacked conformations. Continuous lines are fits of the ST model to the experimental data. (**C**) Re-scaled FECs per base for poly-rA (blue) and poly-rC (red) sequences from Reference (33). Continuous lines are fits of the ST model to the experimental data. In contrast, dashed lines represent the elasticity of the stacked (blue for poly-rA and red for poly-rC) and unstacked (black) conformations. (**D**) Correlation length from the ST model, Eq. (5), as a function of the force for poly-dA, poly-dGdA, poly-rA and polyrC. Statistical uncertainties are shown as shadowed areas. Error bars are the statistical errors from averaging different molecules (Supplementary Table S6).

where *N*_loop_ is the number of bases of each hairpin loop and *x*_*U*_ is the extension of a single unstacked base as given by the TC model (see 'Results' section). The assumption that the bases outside the loop are unstacked is justified by the analysis presented in Figure 2. For the analysis of the ssDNA and ssRNA data from previous works (32,33), presented in Figures 5A,C and 6C, the total number of bases *N* for each studied molecule (poly-dA, mixed, poly-pyrimidine, poly-U, poly-rA and poly-rC) is obtained by imposing the TC model elasticity at forces 30 ≤ *f* ≤ 50 pN. Their re-scaled extension is computed as *x*_*b*_ = *x*_ssDNA_/*N*. For the mixed sequence (Figure 5C), the same approach described by Eq. 2 is followed, subtracting the extension of the bases that do not belong to any poly-purine domain, i.e. that are not bold in the sequence shown in the caption of Figure 5.

### Helix-coil stacking (ST)-model

To analyze the stacking–unstacking transition observed in the experiments, we use a helix-coil type model denoted as the stacking (ST) model. The ssDNA molecule is modeled as a polymer chain of *Nstackable* bases, that can form stacked (S, blue) and unstacked (U, yellow) domains, Figure 4A. The ST model can be mapped onto a one-dimensional Ising chain where each base *i* = 1, 2, .., *N* is represented by a binary variable σ_*i*_. Bases in an S-domain (σ_*i*_ = 1) and those in a U-domain (σ_*i*_ = −1) exhibit different elastic responses.

Two energy parameters define the model (Figure 4A): the (positive) energy gain per stacked base, ε_ST_; and a cooperativity parameter between adjacent domains, γ_ST_. The elastic response of U-domains is modeled using the Thick-Chain (TC) model (Supplementary Section S2), which accounts for steric effects due to the high flexibility of longer U-domains, especially at low salt concentrations. The extension per U-base in the TC model, *x*_*U*_(*f*), is described by parameters *l*_*U*_, *a* and Δ, as previously explained. In contrast, the high rigidity of the shorter S-domains is well described by the semiflexible WLC model (Supplementary Section S2). The extension per base of S-domains, *x*_*S*_(*f*) depends on the persistence length, *p*_*S*_, and the contour length per base, *l*_*S*_ and is obtained by inverting the interpolation formula of the model proposed in Ref. (41). The total extension *X* of the chain is *X* = *N*_*U*_ *x*_*U*_ + *N*_*S*_ *x*_*S*_, where *N*_*U*_ = ∑_*i* = 1, *N*_(1 − σ_*i*_)/2 and *N*_*S*_ = ∑_*i* = 1, *N*_(1 + σ_*i*_)/2 are the total number of unstacked and stacked bases, respectively. The normalized extension per base is *x*_*b*_ = *X*/*N* with *N* = *N*_*S*_ + *N*_*U*_ being the total number of bases. Upon increasing the force *f*, the longer U-domains become energetically favored (*N*_*U*_ increases while *N*_*S*_ decreases), as illustrated in Figure 4A. The energy function of the ST-model reads:


(3) \begin{eqnarray*}
&& E(\lbrace \sigma _i\rbrace ) = -N_{\textrm {S}}(\lbrace \sigma _i\rbrace )\, \Big (\epsilon _{\rm ST}+\int _0^{f}x_{\textrm {S}}(f^{\prime })df^{\prime }\Big )- \nonumber \\ && -N_{\textrm {U}}(\lbrace \sigma _i\rbrace )\int _0^{f}x_{\textrm {U}}(f^{\prime })df^{\prime }-\gamma _{\textrm {ST}}\sum _{i=0}^{N+1}\sigma _i\sigma _{i+1}.
\end{eqnarray*}

The integrals in (3) are the stretching energy contributions per base in the S and U domains. Fixed (Neumann–Neumann) boundary conditions are imposed at the ends, with σ_0_ = σ_*N* + 1_ = −1 for the *non-stackable* bases outside the poly-dA (or poly-dGdA) region. We have solved the free energy *G*(*f*) of the ST-model of Eq. (3) and derived the FECs using $X(f)=-\frac{\partial G}{\partial f}$. Detailed calculations are provided in Supp. Sec. 5.

For *N* = ∞, the free energy difference between the relaxed ssDNA at zero force and the fully unstacked state (given by Eq. (3) with σ_*i*_ = −1, ∀*i*) is (Supplemenatry Section 5.1):


(4) \begin{eqnarray*}
\Delta G_0^{\textrm { }ST} &=& \frac{\epsilon _{\textrm {ST}}}{2}+\frac{1}{\beta }\log \Bigl [\cosh \left(\beta \frac{\epsilon _{\textrm {ST}}}{2}\right)+ \nonumber \\ && +\sqrt{e^{-4\beta \gamma _{\textrm {ST}}}+\sinh ^{2}\left(\beta \frac{\epsilon _{\textrm {ST}}}{2}\right)}\Bigr ],
\end{eqnarray*}

where β = 1/*k*_B_*T*, with *k*_B_ being Boltzmann’s constant and *T* the temperature.

The ST model also allows computing the stacking correlation length, ξ_*ST*_. Starting from any base of the chain in a state σ_*i*_, ξ_*ST*_ is defined as the distance in nucleotides where correlations of σ decay due to thermal fluctuations, i.e. <σ_*i*_σ_*j*_ > ∼exp ((*j* − *i*)/ξ_*ST*_). In the long chain limit (*N* = ∞) the stacking correlation length ξ_*ST*_ is computed as (Supplementary Section 5.1, Eq. S16):


(5) \begin{eqnarray*}
\xi _{\textrm { }ST} =-\left[\log \left(\frac{\cosh \left(\beta A\right)-\sqrt{e^{-4\beta \gamma _{\textrm {ST}}}+\sinh ^2\left(\beta A\right)}}{\cosh \left(\beta A\right)+\sqrt{e^{-4\beta \gamma _{\textrm {ST}}}+\sinh ^2\left(\beta A\right)}}\right)\right]^{-1}, \nonumber\\
\end{eqnarray*}

where *A* is defined as:


(6) \begin{eqnarray*}
A= \frac{\epsilon _{\textrm {ST}}}{2}-\frac{1}{2}\int _0^{f}\Delta x(f^{\prime })df^{\prime },
\end{eqnarray*}

with Δ*x* = *x*_*U*_ − *x*_*S*_. As shown in Eq. (5), ξ_*ST*_ depends on the value of the force, and it is maximum at the force where FECs exhibit a force-shoulder, indicative of a first-order phase transition.

## Results and discussion

We unzip DNA hairpins with optical tweezers and measure the elastic response of poly-dA tracks using DNA hairpin constructs with poly-dA loops of 20 and 40 bases (b) and stems of 15 and 20 bps (*H*_20_ and *H*_40_ in Figure 1A). As a reference, we also study a hairpin sequence with a poly-dT loop of 20 bases (*H*_0_) and a 40b poly-dA loop without stem (*L*_40_), Figure 1A. Stacking is primarily contained in the loop because strands in the stem only contain segments of very short, typically less than four, consecutive purines (see below, Supplementary Figure S1 and Supplementary Table S1), which will be denoted as *non-stackable* sequences. As a comparison we also investigate the elastic response of DNA hairpins with loops of alternating deoxyadenine and deoxyguanine (poly-dGdA) using similar constructs (Figure 6A). Molecules were pulled from their ends using specifically designed DNA handles and a BSO of 48b that links the two handles (39,42) (Figure 1B and 'Methods'). The higher stability to shearing of the dsDNA handle than to the unfolding of the hairpins allows us to obtain the ssDNA molecular extension of the purine loops in a broad range of forces (5 ≤ *f* ≤ 45 pN) (Figure 1C).

### Unstacked elasticity

To measure the FECs of the purine stretches (poly-dA and poly-dGdA) in the loops, we must subtract the contribution of the stem and the BSO to the measured molecular extension (Methods). To derive the elastic response of the *non-stackable* ssDNA sequence in the stem, we have studied the reference hairpin *H*_0_. Figure 2A shows the force *f* versus relative trap position λ, the so-called FDC, in one pulling cycle. The folded hairpin is pulled starting from ∼5pN at a constant loading rate (Figure 2A, green line) until a force is reached (∼40 pN) where the BSO partially detaches (blue oligonucleotide in Figure 1B, bottom), the hairpin unfolds, and a force rip is observed (Figure 2A, top arrow). Upon reaching ∼50 pN the process is reversed, and the force decreases at the same unloading rate (Figure 2A, blue line).

At ∼4 pN, a force jump event is observed (Figure 2A, bottom arrow), where the hairpin refolds, and the BSO rebinds to the right handle (Figure 1B, top). The difference in extension Δλ = λ_*U*_ − λ_*F*_ between the folded and unfolded branches at a certain force (Figure 2A) gives the FEC of the released ssDNA from the hairpin (60 bases) plus the 15 bases released by the BSO (light blue in Figure 1B, and Methods). Figure 2B shows the FEC of the total released ssDNA (75 bases) in 1 M NaCl (brown circles) and 10 mM MgCl_2_ (yellow circles). FECs at the two salt conditions are compatible, in agreement with the 1:100 salt rule of thumb, which states that the screening effect at a given concentration in magnesium equals that at 100 × concentration in sodium (43,44). Results agree with the FECs measured without the BSO at low forces, 4 < *f* < 15 pN. They are also consistent with previous measurements in a poly-pyrimidine sequence (32) (Supplementary Figure S2) confirming that ssDNA from *H*_0_ is fully unstacked.

Figure 2C shows FECs at 10, 50, 100, 500 and 1000 mM NaCl (circles) plotted against the molecular extension per base *x*_ssDNA_/*N*. As a comparison, the results for a 7.2kb ssDNA in glyoxal (9), which prevents secondary structure formation, are also shown (triangles in the first and last panels of Figure 2C). The 7.2 kb results agree with those of *H*_0_, showing that heterogeneous ssDNA sequences lacking many consecutive purines exhibit the same ideal elastic response and can be considered *non-stackable* sequences.

FECs of unstacked ssDNA can be fitted to the Thick Chain (TC) model (45) (Methods, Supplementary Section S2 and Supplementary Figure S4) over three decades of salt concentration (continuous lines in Figure 2B,C). The TC model conceptualizes the ssDNA as a necklace of contour length *L*_*c*_ consisting of oblate disks of diameter 2Δ and spacing *a*. Disks occupy a finite volume to model steric and electrostatic effects (Figure 2B and methods). Fitting the TC model to the data (Supplementary Section 3 and Supplementary Tables S2–S5) we obtain *l*_*U*_ = *L*_*c*_/*N* = 0.652(7) nm (contour length per base), *a* = 0.65(6) nm, and a Debye-like salt dependence for the effective radius, $\Delta =0.40(2)+0.0109(13)/\sqrt{C}$, with *C* the salt concentration in M units. Numbers in parenthesis are the statistical errors in the last digit, obtained from bootstrapping the fitted data points. A similar salt dependence for Δ has been found for RNA poly-U chains (46). The parameters *l*_*U*_ and *a* are salt independent and compatible with each other (orange and black dashed line, central panel of Figure 2C), showing that one disk in the TC model corresponds to a single base of the ssDNA. The value *l*_*U*_ ≃ *a* ≃ 0.65 nm agrees with the reported crystallographic inter-phosphate distance in ssDNA (47). Moreover, half the dsDNA helix diameter (*d*_dsDNA_ ∼2 nm) is compatible with the ssDNA radius predicted by the TC model Δ = *d*_dsDNA_/4 ∼ 0.5 nm (magenta line, central panel of Figure 2C). Figure. 2C (rightmost panel, inset) shows the fitted FECs at different salt concentrations. The TC model predicts a persistence length, *p*_*U*_ = −*a*/log (1 − *a*^2^/(4Δ^2^)),with values ranging from 1.3 nm (10 mM NaCl) to 0.7nm (1M NaCl), consistent with the literature (47,48).

### Poly-dA stacking

The FECs for all constructs in Figure 1A are shown in Figure 3A, averaged over several molecules at 10mM MgCl_2_ (Supplementary Figure S5). If plotted versus the normalized molecular extension *X*_ssDNA_/*N*, hairpins *H*_20_, *H*_40_ and *L*_40_ show a shorter extension upon increasing the poly-dA loop size (Figure 3B). For *H*_40_ and *L*_40_ a nascent shoulder in the FEC is visible around 15pN, a fingerprint of the unstacking transition (33). This shoulder appears as a change in the FEC convexity that deviates from the unstacked elastic response, represented by *x*_*U*_. To extract the contribution of the poly-dA bases in the loop from the FECs, we subtract the elastic contribution of the *non-stackable* bases of the stem and the 15b of the BSO (Supplementary Section S4 and Supplementary Figure S6). The FECs for the loops are shown in Figure 3C, with (*HL*)_40_ being the average of the indistinguishable *H*_40_ and *L*_40_ (Supplementary Figure S7). The unstacking transition is now apparent in the FECs, where the shoulder becomes more prominent for larger loop sizes. These finite-size effects demonstrate that the unstacking transition is cooperative.

To interpret the data, we introduce a helix-coil stacking model (ST model) (Figure 4A and 'Methods' section), where the ssDNA polymer is represented by a chain of *Nstackable* bases, that can be in the stacked (S, blue) and unstacked (U, yellow) state. The elasticity of the stacked bases is given by a Worm-Like Chain (WLC) model, with the contour length *l*_*S*_ and the persistence length *p*_*S*_. In contrast, bases in the unstacked domains follow the TC elasticity as described in the previous section. The Hamiltonian of the model, given by Eq. (3), depends on two energy parameters: the energy gain per stacked base, ε_ST_; and the cooperativity between neighboring bases, γ_ST_. The latter is an interaction energy between adjacent bases that rewards (penalizes) bases being in the same (different) state. As schematically depicted in Figure 4A, each stacked base contributes to the total ssDNA energy with −ε_ST_ whereas neighboring bases in the same (different) state contribute with −γ_ST_ (+γ_ST_).

The model can be analytically solved for a finite chain of *N* bases. Results for the predicted FECs for different *N* are shown in Figure 4B, using the parameters that best fit the experimental results (see below). For small *N* (*N*≲10), the unstacking transition is almost undetectable, while the shoulder in the FEC becomes visible as we approach the thermodynamic limit *N* → ∞. The elasticities of the fully unstacked and fully stacked states are shown as black dashed and dotted lines, respectively. The model also predicts the fraction of stacked bases at a given force, ϕ_*S*_ (Figure 4B, inset), which increases with *N*, saturating for *N* → ∞.

A combined fit of the ST model with four parameters (*l*_*S*_, *p*_*S*_, ε_ST_ and γ_ST_) has been performed for *H*_20_(*N* = 20) and (*HL*)_40_(*N* = 40), giving *l*_*S*_ = 0.386(2) nm, *p*_*S*_ = 9.9(4) nm, ε_ST_ = 0.14(1) kcal/mol and γ_ST_ = 0.86(2) kcal/mol. The inset of Figure 3C compares the FECs predicted by the ST-model using the obtained fitting parameters (black line) with independent experimental data from Ref. (32) for very long poly-dA ssDNA molecules, *N* ∼ 5−40kb (black circles). The inset also compares data from Ref. (32) for a polypyrimidine (poly-pyr) sequence (gray circles) with the TC model prediction for the fully unstacked ssDNA at 1 M NaCl (dashed black line) finding good agreement. Our values, *l*_*S*_ = 0.386(2) nm, *p*_*S*_ = 9.9(4) nm are compatible with those obtained in previous gel electrophoresis studies (*l*_*S*_ ∼ 0.33 nm, *p*_*S*_ ∼ 7.5 nm) (49). *l*_*S*_ is similar to the value for poly-A RNA (33) (*l*_*S*_ = 0.36 nm), and also the step size in B-DNA (0.34nm). While *p*_*S*_ is larger than *p*_*U*_ ∼ 1 nm, it is also lower than for dsDNA (∼50 nm) at comparable salt conditions (50,51).

Regarding the energy parameters, the cost of a domain wall 2γ_ST_ is ten times the stacking energy per base 2γ_ST_ ∼ 10ε_ST_, highlighting the cooperativity of stacking. Interestingly, the values ε_ST_ = 0.14(1) kcal/mol and γ_ST_ = 0.86(2) kcal/mol are similar to those for non-specific secondary structure in ssDNA, ε = 0.18 kcal/mol and γ ∼ 0.61 kcal/mol at 10 mM MgCl_2_ (9), suggesting that secondary structure formation is mainly driven by stacking.

### Salt dependence of base stacking

To further elucidate ssDNA stacking, we have investigated the effect of salt by pulling *H*_40_ at different NaCl concentrations (50, 100, 500 and 1000 mM, Supplementary Figure S8). Figure 5A shows results for *H*_40_ at two selected concentrations (100 and 1000 mM; filled black squares in the middle and right panels). Results for 50 and 500mM are shown in Supplementary Figure S9. We compare these results to those of Ref. (32) on long (*N* = ∞) poly-dA sequences (empty red circles). The left panel also shows data from Ref. (32) for 10 mM, for which we do not have data because *H*_40_ does not refold in the unzipping experiments below 50 mM NaCl. We have performed a simultaneous fit of the ST model combining our data for *N* = 40 with data from Ref. (32) over the various salt conditions.

Fits have been performed by imposing a logarithmic salt dependence for the energy parameters of the model, $\epsilon _{\textrm {ST}}=\epsilon _{\textrm {ST}}^0+m_{\textrm {ST}}^{\epsilon }\log (C)$ and $\gamma _{\textrm {ST}}=\gamma _{\textrm {ST}}^0+m_{\textrm {ST}}^{\gamma }\log (C)$, with *C* the salt concentration in M units and $\epsilon _{\textrm {ST}}^0=0.14(1)$ kcal/mol and $\gamma _{\textrm {ST}}^0=0.86(2)$ kcal/mol the reference values at 1M NaCl. These values have been imposed from the previous fits at the equivalent 10mM MgCl_2_ salt condition (Figure 3C and Supplementary Figure S10). A logarithmic salt dependence is predicted by thermodynamic activity models of diluted ionic solutions and confirmed in studies of DNA and RNA hybridization (9,19–21,52,53). A Debye-like behavior has been assumed for the persistence length of the stacked bases ($p_S=p_{\infty }+A/\sqrt{C}$ (43)) while *l*_*S*_ = 0.386 nm is taken as salt independent (43).

The fitting curves (Figure 5A, continuous lines) reproduce the experimental FECs. Figure 5B shows the salt dependence of ε_ST_ (blue) and γ_ST_ (magenta), with their uncertainties (shadowed bands). We notice that ε_ST_ ∼ 0.11−0.14 kcal/mol ($m_{\textrm {ST}}^{\epsilon }=0.007(2)$ kcal/mol) and *p*_*S*_ ∼ 10−12 nm remain almost constant, whereas γ_ST_ nearly doubles from 10mM to 1M salt concentration ($m_{\textrm {ST}}^{\gamma }=0.065(17)$). Interestingly, 2$m_{\textrm {ST}}^{\gamma }=0.13(3)$ kcal/mol agrees with the salt correction energy parameter per base pair for DNA hybridization in the NN model (0.11 kcal/mol) (19–21). Table 1 shows the fitting parameters obtained with the outlined procedure (procedure I, central column).

**Table 1.** Poly-dA fitting parameters to the ST-model

Relation (C in M units)	Procedure I	Procedure II (kcal/mol, nm)
(C in M units)	(kcal/mol, nm)	(kcal/mol, nm)
$\epsilon _{\textrm {ST}}=\epsilon ^0_{\textrm {ST}}+m^{\epsilon }_{\textrm {ST}}\log \left(C\right)$	$\epsilon ^{0}_{\textrm {ST}}= 0.14(1)$ , $m^{\epsilon }_{\textrm {ST}}=0.004(1)$	$\epsilon ^{0}_{\textrm {ST}}= 0.13(1)$ , $m^{\epsilon }_{\textrm {ST}}=0.006(2)$
$\gamma _{\textrm {ST}}=\gamma ^0_{\textrm {ST}}+m^{\gamma }_{\textrm {ST}}\log \left(C\right)$	$\gamma ^0_{\textrm {ST}}= 0.86(2)$ , $m^{\gamma }_{\textrm {ST}}=0.05(1)$	$\gamma ^0_{\textrm {ST}}= 0.88(3)$ , $m^{\gamma }_{\textrm {ST}}=0.05(2)$
$\gamma _{2}=\gamma ^0_{2}+m_{\gamma _2}\log \left(C\right)$	$\gamma ^0_{2}= 0.5(1)$ , $m_{\gamma _2}=0.08(3)$	$\gamma ^0_{2}= 0.5(2)$ , $m_{\gamma _2}=0.09(3)$
$\gamma _{2}=\gamma ^0_{2}+m^{\gamma }_{\textrm {ST}}\log \left(C\right)$	$\gamma ^0_{2}= 0.43(5)$	
$p_S=p_{S,\infty }+m_p/\sqrt{C}$	*p*_*S*, ∞_ = 9.9(9), *m*_*p*_ = 0.3(1)	*p*_*S*, ∞_ = 4.5(4), *m*_*p*_ = 0.9(5)
*l*_*S*_ = *constant*	*l*_*S*_ = 0.386(2)	*l*_*S*_ = 0.40(1)^1^

^1^Values from procedure I were obtained by fitting the poly-dA data at different salt conditions and ssDNA lengths to the ST-model (single γ). Next, the mixed sequence data is fitted adding the γ2 parameter. Values obtained from procedure II were obtained by simultaneously fitting all sequences and salt concentrations using the γ2-ST model.

We compare our model predictions with previous data for long (*N* → ∞) poly-dA sequences. For *N* = ∞, the stacking free energy Δ*G*_0_, defined as the free energy difference between the relaxed ssDNA at zero force and the fully unstacked state is given by Eq. (4). When βγ_ST_ ∼ 1 we obtain $\Delta G_0\sim \epsilon _{\textrm {ST}}+{\cal O}(e^{-4\beta \gamma _{\textrm {ST}}})\simeq 0.14(3)$ kcal/mol for all salt conditions. This value agrees with the salt independent stacking energy reported for poly-dA sequences, Δ*G*_0_ = 0.159(13) kcal/mol (32), and is close to calorimetric and optical estimates obtained for finite *N* sequences, Δ*G*_0_ = 0.09 − 0.12 kcal/mol (54–59).

The ST model permits us to calculate the stacking correlation length ξ_ST_ versus force *f*, Eq. (5) (Figure 5D at 0.01, 0.1, 1 M NaCl). The stacking correlation length sets the minimum nucleation size that triggers stacked domain growth. It shows a maximum $\xi ^{max}_{\textrm {ST}}$ at $f^{\textrm { }max}_{\textrm { }ST}$, dropping to zero at high forces. $\xi _{\textrm {ST}}^{max}$ is salt dependent varying from 4b at 10 mM to 10b at 1M (Figure 5D, inset). The maximum $\xi _{\textrm {ST}}^{max}$ is a consequence of the first order character of the stacking–unstacking transition. The larger extensional fluctuations in the pulling direction are due to the breathing of the planes of the bases leading to the shoulder observed in the FECs (e.g., Figure 3C). One can prove that the maximum in the correlation length $\xi _{\textrm {ST}}^{max}$ occurs at a force $f^{\textrm { }max}_{\textrm { }ST}$ that depends on ε_*ST*_ and the elasticity of the stacked and unstacked states, $\epsilon_{\textrm {ST}}=\int _{0}^{f^{max}} (x_U(f^{\prime })-x_S(f^{\prime })) df^{\prime }$. In contrast, the value $\xi _{\textrm {ST}}^{max}$ only depends on the cooperativity parameter γ_*ST*_, $\xi _{\textrm {ST}}^{max}=-1/(\log (\tanh (\beta \gamma _{\textrm { }ST}))$. Both $\xi _{\textrm {ST}}^{max}$ and $f^{\textrm { }max}_{\textrm { }ST}$ increase with salt concentration. These results are general predictions of helix-coil models and are derived in Supplementary Section 5.1. Interestingly, our predicted value ξ_ST_(*f* = 0) = 4b matches the minimal nucleation length reported in calorimetry experiments (27). The value of $f^{\textrm{ max}}_{\textrm{ ST}}=14-20$ pN also matches the shoulder observed in the FECs; the larger the $\xi _{\textrm {ST}}^{max}$, the more prominent the shoulder is (Figures 3C and 5A). $f^{\textrm { }max}_{\textrm { }ST}$ also agrees with predictions based on electrostatic tension models (32,60,61) (Supplementary Section 6 and Supplementary Figure S11).

Stacking also occurs between purines and pyrimidines (27). To investigate purine-pyrimidine stacking, we have extended the ST model (Eq. 3) by considering purine-like (*stackable*) and pyrimidine-like (*non-stackable*) domains and introducing a cooperativity parameter γ_2_ at the purine-pyrimidine boundaries (Figure 5B, green boxes in top schematics). The FECs predicted by the γ_2_-ST model (Supplemenatary Section 5C) have been fitted to pulling data from Ref. (32) for mixed sequences containing tracks of 6, 8, 9 consecutive purines (Figure 5C, triangles). We have assumed the logarithmic salt dependence, $\gamma _2=\gamma _2^{0}+m_{\gamma _2}\log {C}$ with the previously determined parameters ε_ST_, γ_ST_, *p*_*S*_, *l*_*S*_, *p*_*U*_, *l*_*U*_. We obtain γ_2_ = 0.5(1) + 0.08(3)log *C* (kcal/mol) which is lower than γ_ST_ (kcal/mol) (green and pink lines in Figure 5B) indicating lower stacking cooperativity between purines and pyrimidines. Interestingly, the salt correction parameters for γ_2_ and γ_ST_ are close (0.065 versus 0.08), indicating similar ion activity effects for stacking. In fact, by imposing the salt correction parameter $m_{\textrm {ST}}^{\gamma }$ to the fit of γ_2_ we get compatible results, $\gamma _2^0=0.43(5)$ kcal/mol. Table 1 shows the parameters obtained by a simultaneous fit of all data to the γ_2_-ST model (procedure II, right column) and agrees with the results of the previous analysis (procedure I, middle column).

### Base-stacking in other purine sequences

To further investigate stacking in ssDNA, we have considered poly-dGdA (alternating dA and dG bases) and poly-dG sequences of different lengths embedded in the same hairpin stems shown in Figure 1. Hairpins *H*_20*GA*_ and *H*_20*G*_ have a stem of 20 bp and a loop of 20 bases, whereas hairpins *H*_40*GA*_ and *H*_40*G*_ have a stem of 15 bp and a loop of 40 bases, so the total number of bases are 60 and 70, respectively. Hairpin sequences are shown in Figure 6A, Supplementary Figure S12 and Supplementary Table S1. We have pulled the four new constructs (Supplementary Figures S12–S14). Results on poly-dGdA sequences (*H*_20GA_ and *H*_40GA_) are shown in Figure 6B as green circles and are compared to poly-dA results. A small deviation from the ideal elastic behavior (dashed line) and a a modest shoulder suggest that stacking is weaker for poly-dGdA than for poly-dA. Fits of the ST model are shown as continuous lines. The weaker stacking in poly-dGdA is reflected in the fitting parameters, which give ε_*ST*_ = 0.02(1) kcal/mol and γ_*ST*_ = 0.67(2) kcal/mol, compared to ε_ST_ = 0.14(1) kcal/mol and γ_ST_ = 0.86(2) kcal/mol for poly-dA, leading to a lower free energy of $\Delta G_0^{ST}=0.07(3)$ kcal/mol (as compared to $\Delta G_0^{ST}=0.14(3)$ for poly-dA). Results for the force-dependent correlation length ξ_*ST*_ are shown in Figure 6D. Compared to poly-dA, the correlation length for poly-dGdA shows a less pronounced maximum at a lower force, 12 pN versus 18pN, which is a consequence of the lower stacking cooperativity (γ_*ST*_) and stability (ε_*ST*_) in poly-dGdA. The lower $\xi ^{max}_{\textrm {ST}}$ aligns with the negligible finite-size effects observed in the FECs of *H*_20GA_ and *H*_40GA_, dark and light green color circles in Figure 6B.

Finally, pulling experiments on the poly-dG constructs lead to non-reproducible FECs that we interpret as due to the formation of compact structures, such as G-quadruplexes, that unfold at forces higher than 40pN. The remarkable kinetic stability of such structures precludes stacking measurements of poly-dG sequences using our method (see Supplementary Figure S14.).

### Stacking of ssRNA

Our study of poly-dA naturally extends to poly-rA sequences, relevant for the tailing of mRNA during the maturation process (62). Poly-rA tails contain hundreds of rA bases that confer a high rigidity to the backbone potentially influencing mRNA translation and gene expression (63). Previous force-spectroscopy measurements in few kilobases homopolymeric ssRNA molecules revealed a stacking–unstacking transition with the characteristic FEC shoulder (33). While no stacking was observed for poly-U, stacking was observed for poly-rA and poly-rC. The poly-U FEC is well described by the ssDNA unstacked elasticity of the TC model, as shown in Supplementary Figure S15. We have analyzed the data of poly-rA and poly-rC from Ref. (33) with our ST-model (*N* = ∞) successfully reproducing the data, Figure 6C. We find that the stacking energy parameter ε_*ST*_ is larger for poly-rA than for poly-dA (0.18 versus 0.14kcal/mol), in agreement with the higher value of the force where the shoulder occurs in the FEC is observed for poly-rA. On the other hand, the cooperativity parameter γ_*ST*_ is lower for poly-rA than for poly-dA (0.5 versus 0.8 kcal/mol), which results in a shorter stacking correlation length, as shown in Figure 6D. Besides, the maximum correlation length is observed at larger forces (∼20–25 pN, Figure 6D), in agreement with the larger stacking–unstacking transition force. In contrast, the free energy of stacking per base of poly-rA at zero force, Eq. 4, is 1.6 times larger than that of poly-dA. For poly-rC, we obtain a lower ε_*ST*_ = 0.13 kcal/mol than that for poly-rA but a similar γ_*ST*_ = 0.4 kcal/mol, leading to a lower correlation length (Figure 6d) and stacking free energy ($\Delta G_0^{ST}=0.20$ kcal/mol for poly-rC vs $\Delta G_0^{ST}=0.25$ kcal/mol for poly-rA).

## Conclusions

Base pairing and stacking are recognized as the main driving forces in NAs folding. While Watson–Crick base pairing is key to modeling specific secondary structures, stacking is less specific and non-local, tending to pile up bases along molecular chains. The cumulative effect of several stacked bases does lead to cooperative and collective effects. Despite their importance, stacking energies in ssNA are poorly known due to their low values, about ∼0.1 kcal/mol per base. Here, we have applied the blocking-splint oligo method to accurately measure the mechanical response of poly-dA tracks of 20–40 bases in a broad range of forces and salt conditions using pulling experiments. A helix-coil model for stacking reproduces the experimentally measured FECs, showing finite-size effects. Such effects are due to the finite stacking correlation length ξ_ST_ ∼ 5−10 b, on the scale of 20–40b of the poly-dA loops studied in the paper. We find that cooperativity increases with salt concentration, doubling from γ_ST_ ∼ 0.5 to ∼0.9 kcal/mol from 10 mM to 1 M NaCl. Cooperativity is ten times larger than the energy parameter ε_ST_ ∼ 0.1 kcal/mol and the stacking free energy per base (Eq. (4)) $\Delta G_0^{ST}\simeq 0.14(3)$ kcal/mol, which are nearly salt independent. These results suggest that cooperativity is salt-dependent, despite that stacking stability remains salt-independent, in agreement with previous results (32). Consequently, the shoulder of the FECs of poly-dA tracks becomes more prominent with salt, while the area between the FECs and the unstacked elastic response remains constant (Supplementary Figure S16). Moreover, the salt correction parameter for DNA hybridization in the NN model (∼0.11 kcal/mol) (19–21) equals twice the salt correction parameter for γ_*ST*_, $2m_{\textrm {ST}}^{\gamma }=0.13(3)$ kcal/mol, suggesting that double helix stability is mainly due to stacking, in agreement with other studies (7,8). Remarkably, the measured elasticities in 10mM MgCl_2_ and 1M NaCl are indistinguishable, indicating that the 1:100 salt rule-of-thumb holds for the stacking–unstacking transition. Therefore, the stacking cooperativity, stability and correlation length can be extrapolated to physiological conditions (64,65), of equivalent ionic strength ∼150−250 mM NaCl: γ_*ST*_ = 0.7 kcal/mol, ε_*ST*_ = 0.14 kcal/mol, ξ_*ST*_ = 7 − 8 b.

We have also studied other purine sequences, such as poly-dGdA tracks of weaker stacking showing a lower ε_*ST*_ , γ_*ST*_ and $\Delta G_0^{ST}$, leading to shorter ξ_*ST*_ at the stacking–unstacking transition force and negligible finite-size effects. Overall, the poly-purine sequences studied present a strong stacking cooperativity with a correlation length of around 4b at zero force. Besides, the formation of stable G-quadruplexes-like structures precludes stacking measurements in poly-dG sequences using our approach. Finally, we have applied the ST model to homopolymeric ssDNA and ssRNA sequences studied in previous works (32,33), finding that stacking energies are larger for ssRNA. In contrast, the cooperativity and stacking correlation length are lower.

Stacking cooperativity is crucial in NAs. In duplex DNA, stacking is responsible for the allosteric effects (66) that propagate long-range interactions in ligand binding, important for regulating gene expression (67). Mechanical models with cooperativity find collective binding affinities of periodicity equal to the helical pitch (68). Cooperativity effects in the form of stacked base triplets have also been observed in overstretched DNA that might be related to the triplets of the genetic code (69). Besides, RNA folds cooperatively into large tertiary structures stabilized by water bridges between phosphates and bases and additional inter-strand stacks. Previous studies of poly-rA RNA molecules (11,59,70) showed that they form double-stranded helices stabilized by stacking, despite being unable to form Watson–Crick base pairs. We generally expect stacking interactions to be more important than hydrogen bonding for cooperativity effects in duplex and ssNA since stacking is the only force that naturally propagates along the phosphate backbone. Our results (γ_*ST*_ > >ε_*ST*_) support the relevance of stacking cooperativity in ssNA in promoting different kinds of structures. The persistence and stacking correlation lengths in ssNA are central parameters for understanding hybridization and assembly of ssNA sequences, a key process for synthetic devices such as DNA origami (15), DNA nanoswitches (16) and synthetic molecular machines (17).

Moreover, the stacking properties of ssDNA regulate the binding affinity of different single-stranded binding proteins involved in replication and recombination (4). The distinct elastic properties of homopolymeric sequences imply different affinities of regulatory proteins that can be characterized using high-throughput techniques such as FRET platforms (71) and electrostatic traps (72). Moreover, the studies of homopolymeric sequences could be used as labels or targets for recognizing specific sequences, such as in immune detection (13). Overall, the distinct behaviors of homopolymeric single-stranded sequences might have been important in codifying specific functionalities in some stages of evolution.

Remarkably, in recent work, we have shown that RNA, but not DNA, exhibits novel properties at cold temperatures below 20ºC attributed to the ribose-water interactions (73). It would be interesting to measure the temperature-dependent stacking in ssRNA and ssDNA to search for differences in the ribose-deoxyribose replacement. Measurements varying the temperature would also allow to determine the entropy, enthalpy and Δ*C*_*p*_ of stacking.

## Supplementary Material

gkae934_Supplemental_File

## Data Availability

The data underlying this article will be shared on reasonable request to the corresponding author.
