# Correction to 'Stacking correlation length in single-stranded DNA'

**DOI:** 10.1093/nar/gkaf724

**Published:** 2025-07-19

This is a correction to: Xavier Viader-Godoy, Maria Manosas, Felix Ritort, Stacking correlation length in single-stranded DNA, *Nucleic Acids Research*, Volume 52, Issue 21, 27 November 2024, Pages 13243–13254, https://doi.org/10.1093/nar/gkae934

The following changes have been made to the originally published manuscript.

In section **Funding**: “[…] the Spanish Research Council Grant [PID2019-111148GB-100][…]” has been emended to read: “[…]the Spanish Research Council Grants [PID2019-111148GB-100 and PID2022-139913NB-I00][…]”.

The emendation has been made to the article.

