# Supplementary material for: Stacking correlation length in single-stranded DNA
Source: Nucleic Acids Res. 2024 Oct 26;52(21):13243–54. doi: 10.1093/nar/gkae934 (PMC11602145; doi:10.1093/nar/gkae934)
Supplement: gkae934_Supplemental_File [file gkae934_supplemental_file.pdf]

Stacking correlation length in single-stranded DNA
Supplementary Information

Xavier Viader-Godoy[1,2], Maria Manosas[1], and Felix Ritort[1]

[1]Small Biosystems Lab, Departament de Física de la Matèria Condensada, Facultat de Física, Universitat de Barcelona, Carrer de Martí i Franquès, 1, 08028 Barcelona, Spain
[2] Dipartimento di Fisica e Astronomia Galileo Galilei, Via Francesco Marzolo, 8, Università degli Studi di Padova, 35131 Padova, Italy.

August 2, 2024

S1 DNA substrates

S1.1 Synthesis

The DNA substrates were prepared by annealing three different oligonucleotides (Figure S1): the *sequence* oligonucleotide where we include the sequence of interest embedded between the two handles (in blue), the blocking splint oligonucleotide (in red) and the complementary handle B oligonucleotide (in green). The sequence of the oligonucleotides included in the different molecular constructs used in the optical tweezers assays are shown in Table S1. The *sequence* oligonucleotide for each construct was purchased (*Merck-Sigma Aldrich*) with a Biotin in the 5'-end and it was labelled with a digoxigenin tailing at the 3-end (Terminal Transferase, *Roche*) before the annealing reaction. After a purification step (*QIA Nucleotide removal kit*), all the oligonucleotides were annealed by starting at a high temperature (70 °C) and decreasing the temperature by steps of 1 °C every minute until room temperature was reached.

S2 Elastic models

S2.1 Worm-like Chain Model

Different polymer models have been proposed to describe the elasticity of nucleic acids [1, 2, 3]. A widely used description is the Worm-Like Chain (WLC) model, which presents the following interpolation formula between the low- and high-force regimes [2]:

$$f(x) = \frac{k_B T}{p_0}\left(\frac{1}{4}\left(1 - \frac{x}{L}\right)^{-2} - \frac{1}{4} + \frac{x}{L}\right), \tag{S1}$$

where k_B is Boltzmann's constant, T is the temperature, x is the polymer extension at a given applied force f, L is the total contour length ($L = N\, l_U$, with N the number of nucleotides and l_U the contour length per nucleotide) and p_0 is the persistence length. Here, we used Equation (S1) to reproduce the elastic behavior of the stacked state (main text, Figs. 3c and 4a,c).

S2.2 Thick Chain Model

The TC model (Fig. S4 and main text, inset of Fig. 2b,) describes the polymer as a chain of discs of radius Δ that are spaced by a distance a, with the polymer total extension given by the contour length L_c. One of the main differences between the TC and the WLC model (or the Freely-Jointed Chain model) is that the former includes steric effects, whereas the later not.

At low forces, the self-avoiding properties of the chain make the force to scale with the extension as $f \sim (x/L_c)^{3/2}$, the so-called Pincus regime. While at high forces, the force goes as $f \sim (1 - x/L_c)^{-2}$, similar to other elastic models such as the WLC or Freely-Jointed Chain. Compared to other descriptions that include exclude volume effects [4, 5], this model has the advantage of presenting a unique analytical interpolated expression which works for all forces [6]:

$$f = \frac{k_\mathrm{B} T}{a\,(1 - \hat{x})} \tanh\left(\frac{k_1 \hat{x}^{3/2} + k_2 \hat{x}^2 + k_3 \hat{x}^3}{1 - \hat{x}}\right), \tag{S2}$$

with $\hat{x} = x/L_c$ and the constants k_1, k_2 and k_3 given by

$$\begin{aligned} k_1^{-1} &= -0.28394 + 0.76441\Delta/a + 0.31858\Delta^2/a^2 \\ k_2^{-1} &= +0.15989 - 0.50503\Delta/a - 0.20636\Delta^2/a^2 \\ k_3^{-1} &= -0.34984 + 1.23330\Delta/a + 0.58697\Delta^2/a^2. \end{aligned} \tag{S3}$$

Interestingly, the model can be compared with the WLC model by computing the polymer persistence length using the following relation:

$$p = \frac{-a}{\log\left(1 - \frac{a^2}{4\Delta^2}\right)}. \tag{S4}$$

S3 Fits to the TC model

The unstacked elasticity is modelled by the TC model (Fig. S4 and main text, inset of Fig. 2b and). The experimental FECs corresponding to *unstacked* sequences (H_0 and 7.2 kbp from Ref. [7]) were fitted along the force range $1 \le f \le 25$ pN. The fits of m FECs, each one with n_i experimental points (x_{ij}, f_{ij}) were performed by minimizing the χ^2 function, defined as:

$$\chi^2 = \langle n_i \rangle \sum_{i=1}^{m} \sum_{j=1}^{n_i} w_i \left(\frac{x_{ij} - x(f_{ij})}{\sigma_{ij}} \right)^2, \tag{S5}$$

where $w_i = 1/n_i$, with N being the total number of points $N = \sum_{i=1}^{m} n_i$, σ_{ij} is the error of x_{ij} and $\langle n_i \rangle$ is the average of points per molecule. $x(f_{ij})$ is obtained from Eq. S2 at $f = f_{ij}$. Eq. S5 ensures that all FECs are equally weighted.

Several scenarios regarding the salt dependence of the model parameters a, Δ and l_U were tested. In order to compare them, it is convenient to define the following quantities, the reduced χ^2 and the Akaike Information Criterion. These quantities allow the comparison between models with different number of parameters, p. The reduced χ^2 reads as:

$$\chi^2_\nu = \frac{\chi^2}{\nu} \tag{S6}$$

where ν is the degrees of freedom of the system, $\nu = N - p$, with N being the number of experimental points. On the other hand, the Akaike Information Criterion is defined as:

$$AIC = N \log \left(\chi^2_\nu \right) + 2p. \tag{S7}$$

Since the number of experimental points of the 7 FECs fitted is $N = 126$ $(1 \le f \le 25$ pN), we have neglected the term for AIC accounting for the finite-size of the sample. The best fitted model is the one with the smallest AIC and with $\chi^2_\nu \sim 1$ ($\chi^2_\nu << 0$ ($\chi^2_\nu >> 1$) are considered over(under)fitting of the model). Tables S2-S5 show the best-fitting parameters obtained for the TC model imposing several conditions. The errors have been obtained by bootstrapping the data points with $N = 500$.

S3.1 Fixed a, l_U and Debye-like salt dependence for Δ

The TC model best-fitting parameters, obtained by simultaneously fitting all salt-conditions and sequences, imposing a fixed a and l_U independently of the salt and sequence and the following salt dependence for Δ: $\Delta = \Delta_0 + m_\Delta/\sqrt{C}$, are: $a = 0.65 \pm 0.06$ nm, $l_U = 0.652 \pm 0.007$ nm, $\Delta_0 = 0.41 \pm 0.02$ nm, $m_\Delta = 0.011 \pm 0.001$ nm. Errors are obtained by bootstrapping (N=500) the experimental points. The fit has a $\chi^2 = 3.44\text{nm}^2$, $\chi^2_\nu = 0.284\text{nm}^2$ and $AIC = -149$. AIC is the lowest of all the attempted fits, while $\chi_\nu \sim 1$, rendering it the best model among all tried ones.

S4 Salt-dependence of the elasticity of the 15bp dsDNA segment

The 15bp dsDNA segment formed by the hybridization of the 5-end of the blocking splint oligonucleotide and the central part of the handle B (Figure S2) is unwound at large forces, leading to the unfolding of the DNA construct. In order to extract the ssDNA FEC using the two branches method (See Methods), we characterize the elasticity of the 15bp segment using the Worm-Like Chain model. We combine the elastic parameters at 1M NaCl concentration obtained for two-short 29bp dsDNA segments [8] and the salt dependence of dsDNA from Ref. [9], giving: $l = 0.34$ nm/bp and $P = 2.44 + \frac{0.0324}{C}\left(\frac{7.2}{5.5}\right)^2$, with C being the salt concentration in M. As can be seen in Fig. S6, the salt corrections on the extension of the handles is always below to ~ 1.5 nm, corresponding to a maximum error ~ 0.01 nm in the extension per nucleotide, x_b, of the molecules studied (ranging from 55 to 85 nucleotides).

S5 ST-Models

In the stacking (ST) model, the polymer is represented by a chain of N *stackable* bases, which can be part of a stacked domain (S, $\sigma_i = 1$) or an unstacked domain (U, $\sigma_i = -1$), each one with a specific elastic response. The extension per U-base, $x_U(f)$, is given by the TC model with the parameters L_c, a, and Δ reported in the main text (Main section Unstacked Elasticity). The extension per S-base, $x_S(f)$, is described by the semiflexible WLC model with two parameters: the persistence length p_S, and the contour length per base l_S.

The ST-model is characterized by two energy parameters (main text, Fig. 3d): the (positive) energy gain per stacked base, ϵ_{ST}; and the cooperativity between adjacent domains, γ_{ST}. The latter represents the reward (or penalization) energy associated with adjacent bases that are in the same (or different) state. The ST-model Hamiltonian reads:

$$H(\{\sigma_i\}) = -N_{\mathrm{S}}(\{\sigma_i\}) \left(\epsilon_{\mathrm{ST}} + \int_0^f x_{\mathrm{S}}(f')df'\right) - \\ -N_{\mathrm{U}}(\{\sigma_i\}) \int_0^f x_{\mathrm{U}}(f')df' - \gamma_{\mathrm{ST}} \sum_{i=0}^{N} \sigma_i \sigma_{i+1}, \tag{S8}$$

with $N_S = \frac{1}{2}\sum_i (1+\sigma_i)$ and $N_U = \frac{1}{2}\sum_i (1-\sigma_i)$. The Hamiltonian, given by Eq. (S8), can be written as:

$$\mathcal{H} = -A \sum_i^N \sigma_i - B \sum_i^N \sigma_i \sigma_{i+1} - C, \tag{S9}$$

with

$$A = \frac{\epsilon_{\mathrm{ST}}}{2} - \frac{1}{2}\int_0^f \Delta x(f')df', \tag{S10}$$

$$B = \gamma_{\mathrm{ST}}, \tag{S11}$$

$$C = N \int_0^f \hat{x}(f')df' + D, \tag{S12}$$

(S13)

where $\Delta x = x_U - x_S$, $\hat{x} = \frac{x_U + x_S}{2}$ and D is a constant, and therefore will not be considered in the calculations below.

S5.1 Infinite model

When considering an infinite system ($N \to \infty$, periodic boundary conditions), the partition function Z, associated to the Hamiltonian given by Eq.(S9), can be written as:

$$Z = \sum_{\{\sigma_i\}} e^{-\beta\mathcal{H}(\{\sigma_i\})} = \sum_{\sigma_1} V^N \sigma_1\sigma_1 = tr\left(V^N\right), \tag{S14}$$

where tr is the trace and V is the transfer matrix given by:

$$V = e^{\beta C/N} \begin{pmatrix} e^{\beta(A+B)} & e^{-\beta B} \\ e^{-\beta B} & e^{-\beta(A-B)} \end{pmatrix}. \tag{S15}$$

By diagonalizing V, we find its eigenvalues:

$$\begin{aligned} \lambda_{\pm} &= e^{\beta C/N} e^{\beta B} \cdot \\ &\cdot \left[\cosh(\beta A) \pm \sqrt{e^{-4\beta B} + \sinh^2(\beta A)}\right]. \end{aligned} \tag{S16}$$

The partition function, Eq. (S14), can then be written as:

$$Z_N = \lambda_+^N \left[1 + \left(\frac{\lambda_-}{\lambda_+}\right)^N\right], \tag{S17}$$

which, in the thermodynamic limit $N \to \infty$ ($\lambda_- < \lambda_+$), leads to:

$$Z_N = \lambda_+^N. \tag{S18}$$

To obtain the correlation length, we compute the correlation between spins in the ith and jth position in the chain as:

$$\langle\sigma_i\sigma_j\rangle = \frac{1}{Z_N} \sum_{\{\sigma\}} \sigma_i\sigma_j e^{-\beta\mathcal{H}(\{\sigma\})}. \tag{S19}$$

Using Eq. (S17) and taking the thermodynamic limit $N \to \infty$ using $\lambda_+ > \lambda_-$, the latter can be written as:

$$\langle\sigma_i\sigma_j\rangle = \frac{\lambda_+^{N-(j-i)}\lambda_-^{(j-i)} + \lambda_+^{(j-i)}\lambda_-^{N-(j-i)}}{\lambda_+^N + \lambda_-^N} \approx \left(\frac{\lambda_-}{\lambda_+}\right)^{(j-i)}. \tag{S20}$$

To extract the correlation length we re-write Eq. (S20) in the form of $\langle \sigma_i \sigma_j \rangle = e^{-r/\xi}$, with $r = |j - i|$:

$$\langle \sigma_i \sigma_j \rangle = e^{r \log\left(\frac{\lambda_-}{\lambda_+}\right)^{(j-i)}}, \tag{S21}$$

which allows us to identify the correlation length as

$$\xi = -\frac{1}{\log\left(\frac{\lambda_-}{\lambda_+}\right)}. \tag{S22}$$

Using Eq. (S16), the latter can be written as:

$$\xi = -\log\left(\frac{\cosh(\beta A) - \sqrt{e^{-4\beta B} + \sinh^2(\beta A)}}{\cosh(\beta A) + \sqrt{e^{-4\beta B} + \sinh^2(\beta A)}}\right)^{-1}. \tag{S23}$$

We can find analytical expressions for the maximum in correlation length, ξ_{ST}^{max} and the value of force at which it happens, f_{ST}^{max}. To do so, we derive with respect to force Eq. S23 and impose it to be zero, obtaining

$$y'x = yx', \tag{S24}$$

where x' and y' are the force derivatives of $x = \cosh(\beta A)$ and $y = \sqrt{e^{-4\beta B} + \sinh^2(\beta A)}$. These derivatives are related between them: $y' = x\,x'/y$, for all forces, leading to $y'y = x'x$. This implies that y^2 and x^2 are equal except for a constant, $y^2 = x^2 + e^{-4\beta B} - 1$. Substituting $y' = x\,x'/y$ to Eq. S24, it can be seen that the condition

$$x'x^2 = x'y^2, \tag{S25}$$

can only be true if and only if $x' = 0$, which leads to $A(f_{\mathrm{ST}}^{max}) = \frac{\epsilon_{\mathrm{ST}}}{2} - \frac{1}{2}\int_0^{f_{\mathrm{ST}}^{max}} \Delta x(f')df' = 0$. Therefore, the maximum in correlation length only depends on the stacking energy per nucleotide,

$$\epsilon_{\mathrm{ST}} = \int_0^{f_{\mathrm{ST}}^{max}} \Delta x(f')df, \tag{S26}$$

while the value taken by the stacking correlation length at f_{ST}^{max} is given by

$$\xi_{ST}^{max} = -\log\left(\frac{1+e^{-2\beta\gamma_{ST}}}{1-e^{-2\beta\gamma_{ST}}}\right) = \frac{-1}{\log\left(\tanh\beta\gamma_{ST}\right)}, \tag{S27}$$

which only depends on γ_{ST}. Note that ξ_{ST} has no dimensions and corresponds to the number of bases required to move in the chain, from a starting base i in the state σ_i, to have the correlation between spins $\langle\sigma_i\sigma_{i+\xi_{ST}}\rangle = 1/e$.

S5.2 Finite ST-model

For modeling a finite-size N-bases *stackable* domain, we use the Hamiltonian given by Eq. (S9) considering $N+2$ bases, where the $i=0$ and $i=N+1$ are fixed at the unstacked state $\sigma=-1$ (Neumann-Neumann boundary conditions). In this scenario, the partition function can be written as:

$$Z = \sum_{\sigma_1=\pm1}\cdots\sum_{\sigma_N=\pm1} e^{\beta C} e^{\beta(A/2-B)\sigma_1} e^{(\beta A/2)(\sigma_1+\sigma_2)+\beta B(\sigma_1\sigma_2)}\cdots$$
$$\cdots e^{(\beta A/2)(\sigma_{N-1}+\sigma_N)+\beta B(\sigma_{N-1}\sigma_N)} e^{\beta(A/2-B)\sigma_N}. \tag{S28}$$

This expression can be further simplified by using the transfer matrix, Eq. (S15), as:

$$Z = \sum_{\sigma_1=\pm1}\sum_{\sigma_N=\pm1} e^{\beta C/N} e^{\beta(A/2-B)\sigma_1}\langle\sigma_1|V^{N-1}|\sigma_N\rangle e^{\beta(A/2-B)\sigma_N}. \tag{S29}$$

We can write V as a function of the diagonalized matrix V' as $V = S^{-1}V'S$, where S is the transformation matrix containing the eigenvectors of V, $\vec{v}_+$ and $\vec{v}_-$. As V is a symmetric matrix, the obtained eigenvectors will be orthonormal. Solving for the eigenvector corresponding to λ_+, we obtain the following orthonormal eigenvectors:

$$\vec{e}_+ = \frac{1}{\sqrt{1+k^2}}\begin{pmatrix}k\\1\end{pmatrix}, \tag{S30}$$

$$\vec{e}_- = \frac{1}{\sqrt{1+k^2}}\begin{pmatrix}1\\-k\end{pmatrix}, \tag{S31}$$

with $k = -e^{-2\beta B}/\left[e^{\beta A}\cosh\left(\beta A\right)+\sqrt{e^{-4\beta B}+\sinh^2\left(\beta A\right)}\right]$.

The transformation matrix then verifies:

$$S = S^{-1} = \frac{1}{\sqrt{1+k^2}} \begin{pmatrix} k & 1 \\ 1 & -k \end{pmatrix}, \tag{S32}$$

which allows us to write the partition function of the system as:

$$\begin{aligned} Z =& \frac{\lambda_+^{N-1}}{1+k^2} \left\{ e^{\beta(C/N+A-2B)} \left[k^2 + \left(\frac{\lambda_-}{\lambda_+}\right)^{N-1} \right] + \right. \\ & \left. + e^{\beta(C/N+2B-A)} \left[1 + k^2 \left(\frac{\lambda_-}{\lambda_+}\right)^{N-1} \right] + 2k \left[1 - \left(\frac{\lambda_-}{\lambda_+}\right)^{N-1} \right] \right\}. \end{aligned} \tag{S33}$$

From the partition function, the free energy is directly computed as:

$$G = -k_\mathrm{B} T \log Z. \tag{S34}$$

The extension as a function of the force can be obtained analytically from the free energy as:

$$x = -\frac{\partial G}{\partial f}. \tag{S35}$$

S5.3 Purine-pyrimidine model

When considering a different coupling energy between *stackable* (e.g. poly-dA) bases and *non-stackable* (e.g. pyrimidine) bases, γ_2, the partition function of the system can be written as in Eq. S33, by changing the B terms due to the boundaries of the system by the new interaction energy, γ_2:

$$\begin{aligned} Z =& \frac{\lambda_+^{N-1}}{1+k^2} \left\{ e^{\beta(C/N+A-2\gamma_2)} \left[k^2 + \left(\frac{\lambda_-}{\lambda_+}\right)^{N-1} \right] + \right. \\ & \left. + e^{\beta(C/N+2\gamma_2-A)} \left[1 + k^2 \left(\frac{\lambda_-}{\lambda_+}\right)^{N-1} \right] + 2k \left[1 - \left(\frac{\lambda_-}{\lambda_+}\right)^{N-1} \right] \right\}. \end{aligned} \tag{S36}$$

From the partition function, the free energy of the system and its extension can be then derived from Eqs. S34 and S35.

S6 Electrostatic tension of poly-dA stacking

The stacking-unstacking transition in poly-dA has been used, in previous works [10], to quantify the effect of the intrinsic electrostatic tension of stacked DNA, f_{el}. The structural transition of stacked DNA occurs at a characteristic force $f_{tot} = f_{app} + f_{el}$, where f_{app} is the externally applied force at which the transition occurs and f_{el} is an electrostatic tension characterized by a a charge distance in the chain a, a Bjerrum length l_B and a salt dependent Debye length κ^{-1}. f_{el} has been described by several electrostatic theories [11, 12].

By definition, f_{app} corresponds to the stacking transition point where $d^2f/dx^2 = 0$, i.e. the inflection point of the FEC. In Fig. S11 we show f_{app} as a function of the salt concentration C (in mM). The results obtained for f_{app} from Ref. [10] are shown as magenta squares. The red continuous line shows f_{app} computed using the ST-model with the best-fitting parameters at each salt concentration for poly-dA. As a comparison, we also show, as a blue continuous line, the salt dependence of the force f_{max} at which the correlation length is maximum (Main text, Fig. 5d). Interestingly, f_{app} and f_{max} are similar, even though the first method systematically reports a shift in force (about ~ 1.5 pN larger), for all salt concentrations. Both f_{app} and f_{max} are fitted to the Netz [11] and Manning [12] electrostatic models, using two fitting parameters, a and f_{tot}. The obtained values (reported in the key of Fig. S11) are compatible to those reported in Ref. [10], albeit showing a higher intrinsic electrostatic tension f_{tot}, for both cases.

S7 Error determination

In order to assess the errors associated to the model fitting parameters, we have used a bootstrap method. Briefly, fits of the model to the experimental FECs are performed 100-500 times using a randomly chosen subset of experimental data points. The values of the model parameters, ϵ and γ are estimated as the average value from the different fits with an error that is set equal to the standard deviation.

To validate this method, we have computed an upper limit for the error of ϵ and γ based on the error of the extension X, δX. δX takes into account the experimental error on the measured extension λ as well as that of the different contributions needed to compute x_b [main text Eqs. (1) and (2)]. To do so, we have fitted the poly-dA data shown in main Figure 3c, considering the upper and lower limits of the extension: $X + \delta X$ and $X - \delta X$. The difference in the fitting parameters obtained in the two limit cases gives an upper estimate for the error, which leads to $\Delta\epsilon = 0.036$ kcal/mol and $\Delta\gamma = 0.097$ kcal/mol. The value of this maximum error estimation is on the order of the error obtained with the bootstrap method, which validates the approach used to determine the errors. The obtained errors are then propagated into both free energy of formation of stacking per base, ΔG_0^{ST}, and the correlation length, ξ, considering the fitting parameters as independent variables.

In Table S6, we report the number of molecules and the number of cycles analyzed, as well as the standard deviation and error in the measured extension (at a reference force of 20 pN), for each studied molecule and experimental condition. The standard deviation of the extension obtained for a single-molecule over N_{cycles}, and for the average of N_{mol} molecules is of the same order of magnitude of the instrumental precision ($\sim$ 1 nm).

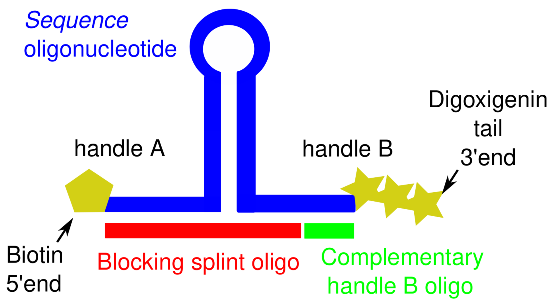

Figure S1: **Schematics of the hairpin design.** The different oligonucleotides forming the DNA construct used for the optical tweezers experiments are shown in different colors. The corresponding sequences are shown in Table S1.

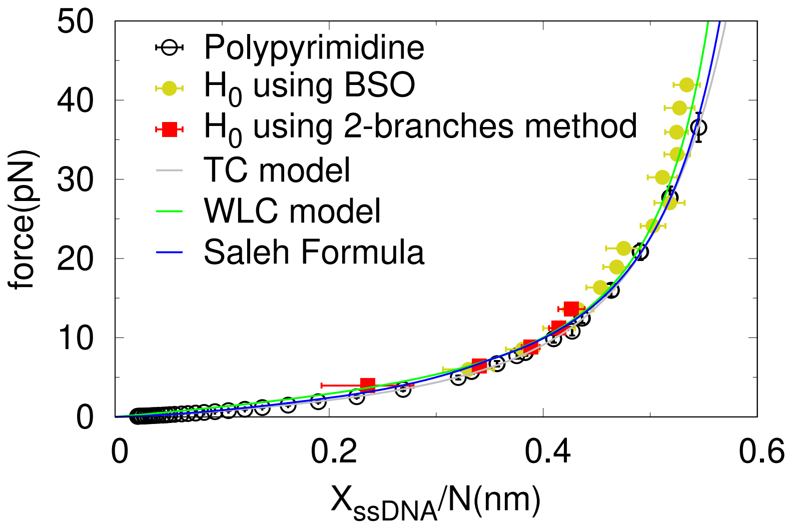

Figure S2: **FECs of H_0 and poly-pyrimidine comparison.** FECs of H_0 obtained with (yellow, circles) and without (red squares) the Blocking Splint Oligonucleotide (BSO), using the two branches method. For comparison, the FEC of a polypyrimidine sequence from Ref. [10] (empty black circles), rescaled as described in Methods, is also shown. As a reference, the predicted FEC using the TC model, WLC model and Saleh Formula [7] are represented using grey, green and blue lines. All experimental results shown are compatible among them, proving the validity of the BSO method and suggesting that H_0 does not experience any significant stacking, i.e. it presents an elasticity equivalent to that of polypyrimidine sequences.

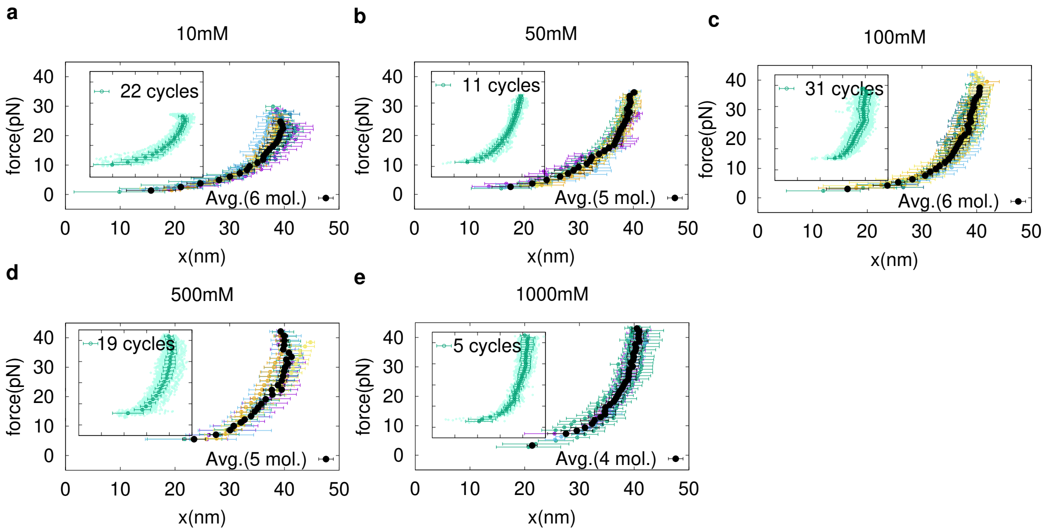

Figure S3: **FECs of H_0 for varying NaCl concentration. a.** FECs for the H_0 construct at 10 mM NaCl for 6 molecules (empty circles) and the resulting averaged curve (black circles). Inset: averaged FEC for one molecule, over 22 pulling cycles. **b.**, **c.**, **d.** and **e.** Same as in **a.** but for 50 mM (5 molecules), 100 mM (6 molecules), 500 mM (5 molecules) and 1000 mM (4 molecules) NaCl data, respectively. To illustrate the dispersion of the measured extension between cycles the standard error is shown in the insets for one of the molecules. Error bars in the main figures are the standard error of the mean over the different molecules studied at each condition.

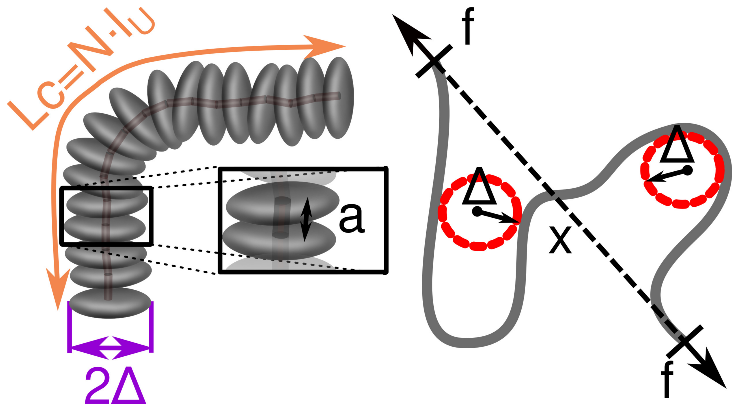

Figure S4: **Schematic depiction of the Thick Chain model.** (Left) The three parameters that characterize the model are the disk effective radius Δ, their spacing a and the total contour length, L_c (each of the N units with a contour length of l_U). (Right) Sketch of the curve which is a viable centreline for a tube of thickness Δ, which shows an extension x under an externally applied force f. The radii of the circles through any triplet of points are not smaller than Δ (Figure adapted from Ref. [13]).

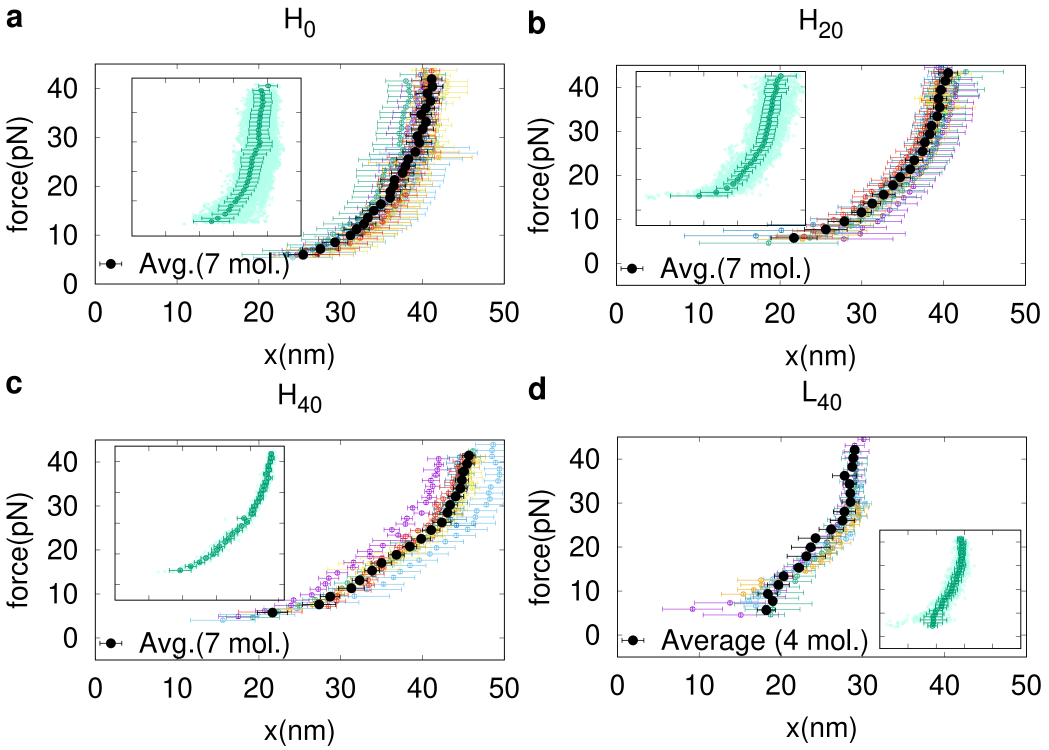

Figure S5: **FECs of H_0, H_{20}, H_{40} and L_{40} at 10 mM $MgCl_2$ a.** FECs for the H_0 construct for 7 different molecules (empty circles) and the resulting averaged curve (black circles). The inset shows the FEC for one of the molecules averaged over 22 pulling cycles. **b.** FECs for the H_{20} construct for 7 different molecules (empty circles) and their average (black circles). The inset shows the FEC for one of the molecules averaged over 41 pulling cycles. **c.** FECs for the H_{40} construct for 7 different molecules (empty circles) and the resulting averaged curve (black circles). The inset shows the FEC for one of the molecules averaged over 3 pulling cycles. **d.** FECs for the L_{40} construct for 4 different molecules (empty circles) and the resulting averaged curve (black circles). The inset shows the FEC for one of the molecules averaged over 7 pulling cycles. Error bars in the main figures are the standard error of the mean over the different molecules studied at each condition.

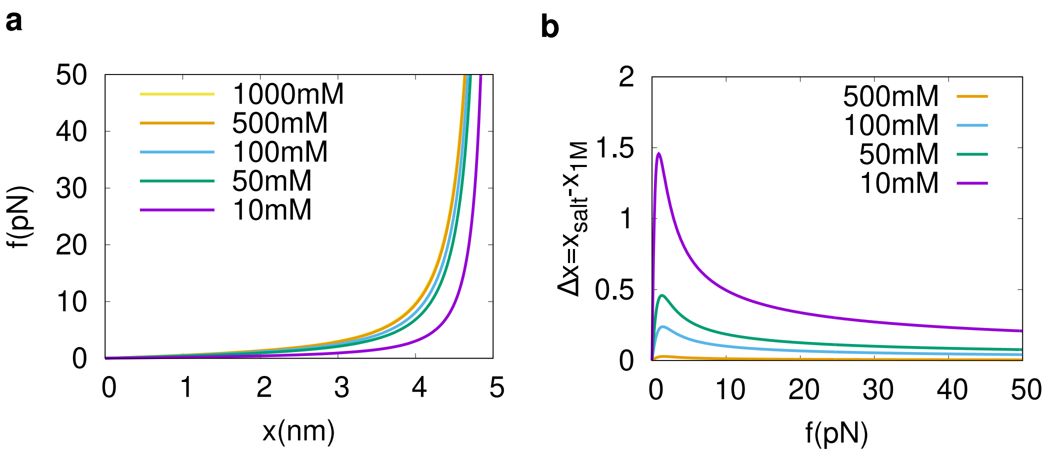

Figure S6: **Effect of the 15bp DNA segment elasticity on the ssDNA FECs. a.** FECs of the 15bp DNA segment at varying salt concentrations. **b** Difference in extension as a function of the force Δx for each salt concentration with respect that of 1M NaCl concentration.

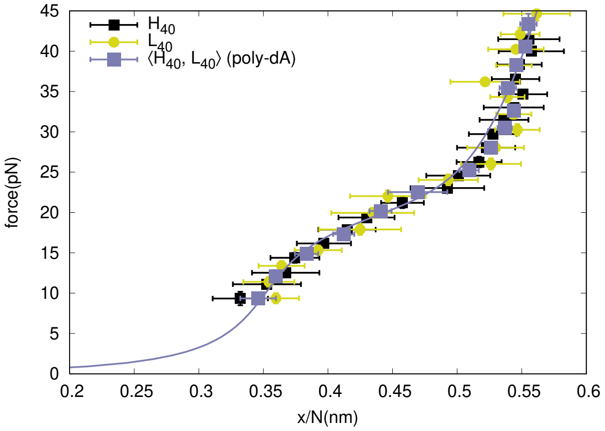

Figure S7: **Comparison of H_{40} and L_{40}.** FECs corresponding to the 40 bases poly-dA loop obtained from the measured elasticity of the H_{40} (black squares) and L_{40} (yelow circles) constructs as explained in S1. The average of the two FECs curves is shown in blue (squares), as well as the fit obtained in the main text (Section Poly-dA stacking, continuous line). The error bars are the error of the mean obtained from averaging over different molecules.

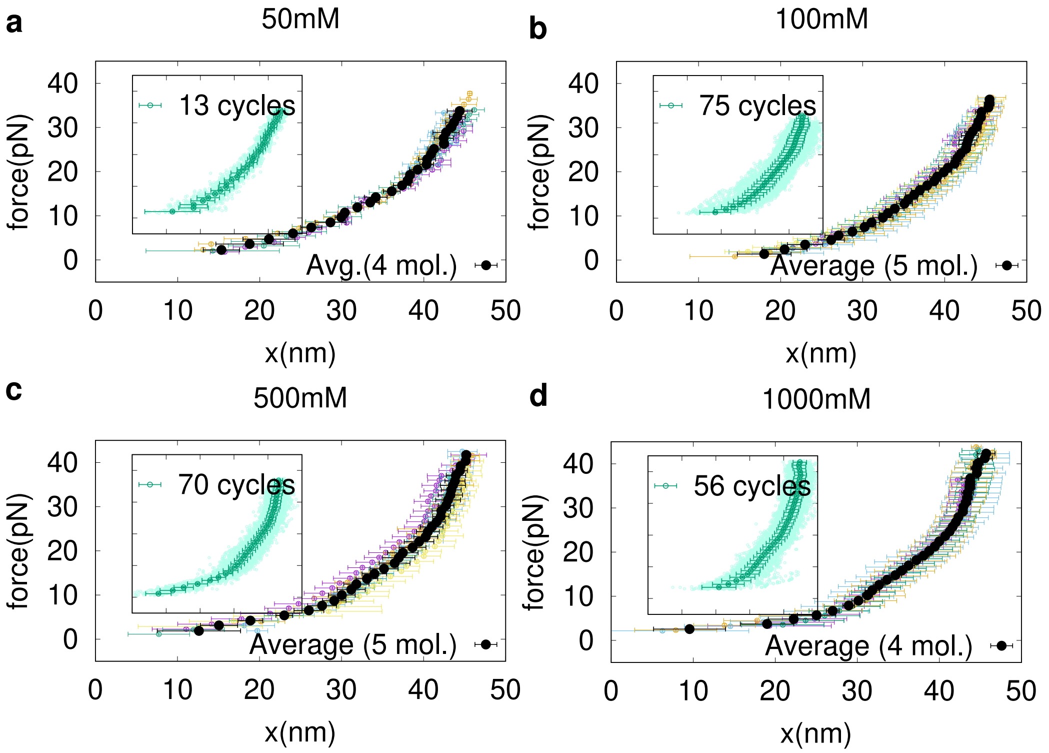

Figure S8: **FECs of H_{40} for varying NaCl concentrations. a.** FECs for the H_{40} construct at 50 mM NaCl for 4 different molecules (empty circles) and the resulting averaged curve (black circles). The inset shows the FEC for one of the molecules averaged over 13 pulling cycles. **b.** FECs for the H_{40} construct at 100 mM NaCl for 5 different molecules (empty circles) and the resulting averaged curve (black circles). The inset shows the FEC for one of the molecules averaged over 75 pulling cycles. **c.** FECs for the H_{40} construct at 500 mM NaCl for 5 different molecules (empty circles) and the resulting averaged curve (black circles). The inset shows the FEC for one of the molecules averaged over 70 pulling cycles. **d.** FECs for the H_{40} construct at 1000 mM NaCl for 4 different molecules (empty circles) and the resulting averaged curve (black circles). The inset shows the FEC for one of the molecules averaged over 56 pulling cycles. Error bars are statistical errors.

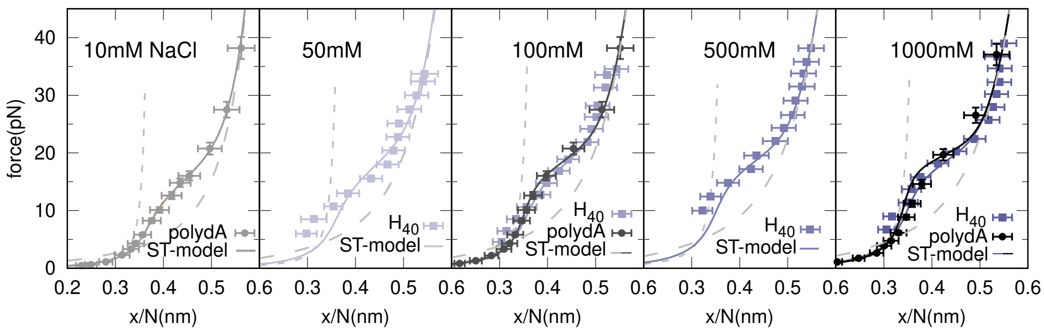

Figure S9: **Fits of the ST-model to H_{40} data and poly-dA data from Ref. [10]**.FECs showing the extension per base, x/N, of the poly-dA regions at 10, 50, 100, 500 and 1000 mM NaCl. Solid curves correspond to the best-fit obtained using the finite (for H_{40}) and infinite (for long poly-dA from Ref. [10]) ST-model. The error bars are statistical errors associated to the average between different molecules.

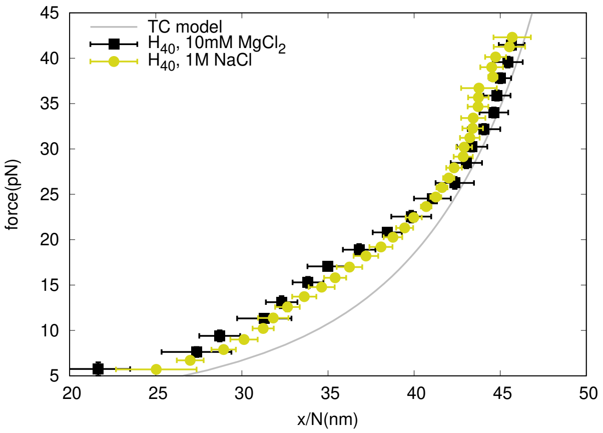

Figure S10: **FECs of H_{40} at 1M NaCl and 10 mM $MgCl_2$.** FECs for the H_{40} construct measured at 10 mM $MgCl_2$ (black squares) and 1M NaCl (yelow circles). The two curves practically overlap as expected by the 1-100 rule [14]. As a reference, the unstacked elasticity is represented using the TC model (with the parameters obtained by fitting the H_0 data). The difference between the unstacked elasticity and the H_{40} elasticity reveals the presence of stacking. The error bars are statistical errors associated to the average between different molecules.

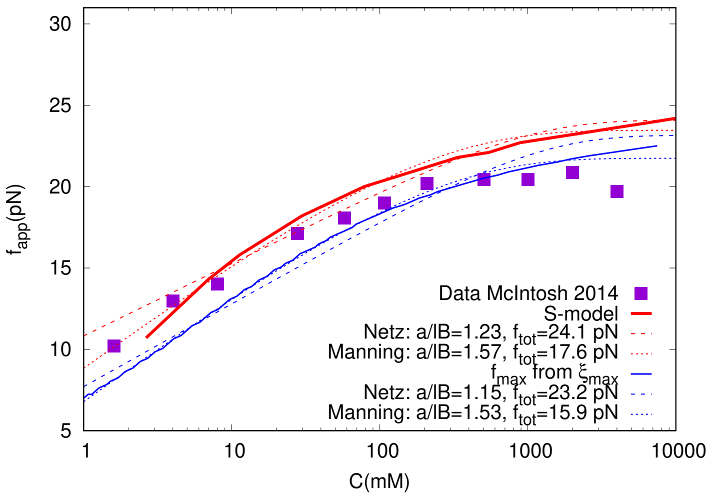

Figure S11: **Applied force at the unstacking transition as a function of ionic strength.** Magenta squares are the data from Ref. [10]. The red continuous line corresponds to the ST-model prediction, which is fitted to the Netz[11] and Manning[12] models (red dashed and point lines, respectively). The blue line shows the predicted applied force from the ST-model considering the force, f_{max}, where the maximum of the correlation length, ξ_{max}, is found (See Fig.5d. Main Text), which is fitted to the Netz[11] and Manning[12] models (blue dashed and point lines, respectively).

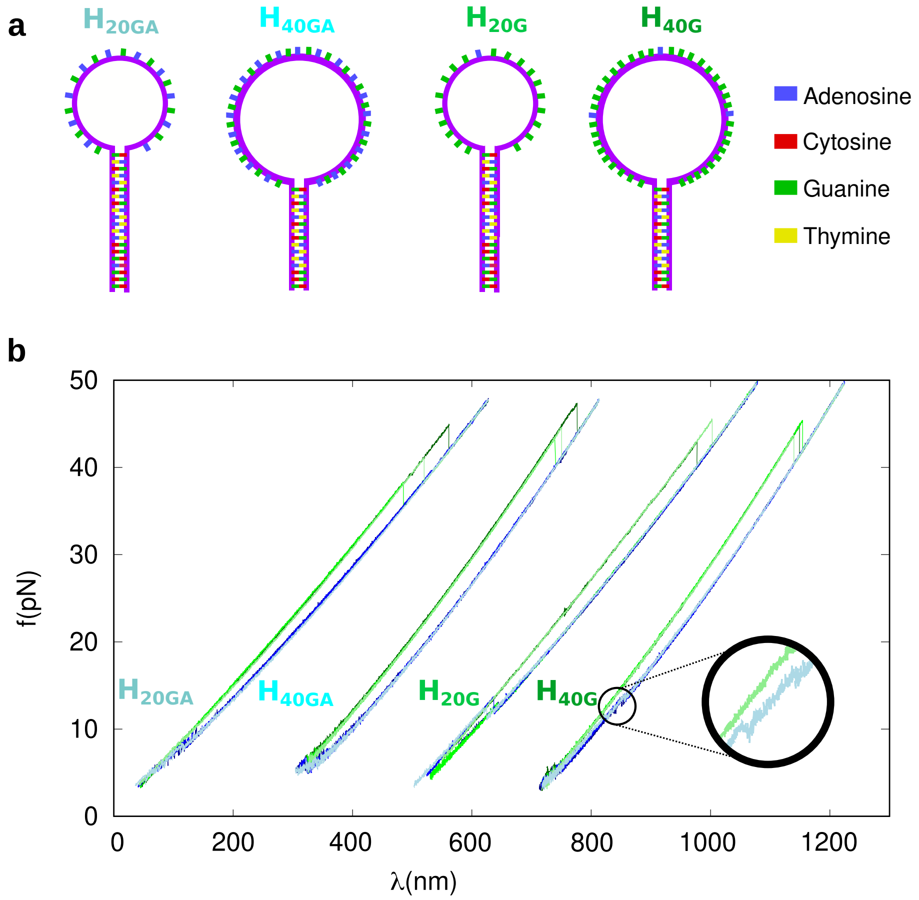

Figure S12: **poly-dGdA and poly-dG hairpins and FDCs. a.** Schematic depictions of the four hairpins containing dG and a combination of dG and dA in the loop. **b.** Typical force-distance curves (FDCs) for the 4 hairpins above depicted. In green (blue), the unfolding (folding) curves. Typical force-distance curve (FDC) for the molecules shown in panel (a). Since all molecules share the same blocking-splint oligonucleotide, they all unfold at a similar force of ~ 45 pN, while their refolding strongly depends on the length loop and sequence, while their refolding depends on the length and sequence of the loop. The zoom at the bottom right shows some intermediate structures appearing in the H_{40G} refolding.

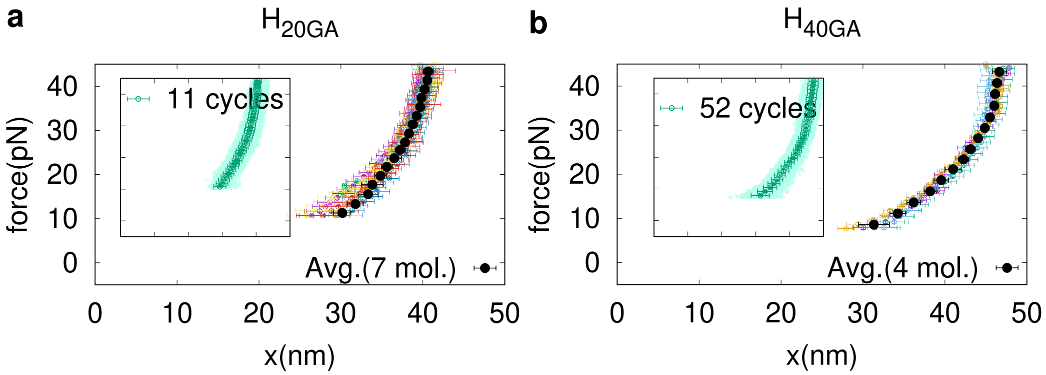

Figure S13: **FECs of H_{20GA} and H_{40GA}** **a.** FECs for the H_{20GA} construct at 10 mM $MgCl_2$ for 7 different molecules (empty circles) and the resulting averaged curve (black circles). The inset shows the FEC for one of the molecules averaged over 11 pulling cycles. **b.** FECs for the H_{40GA} construct at 10 mM $MgCl_2$ for 4 different molecules (empty circles) and the resulting averaged curve (black circles). The inset shows the FEC for one of the molecules averaged over 52 pulling cycles. Error bars are statistical errors.

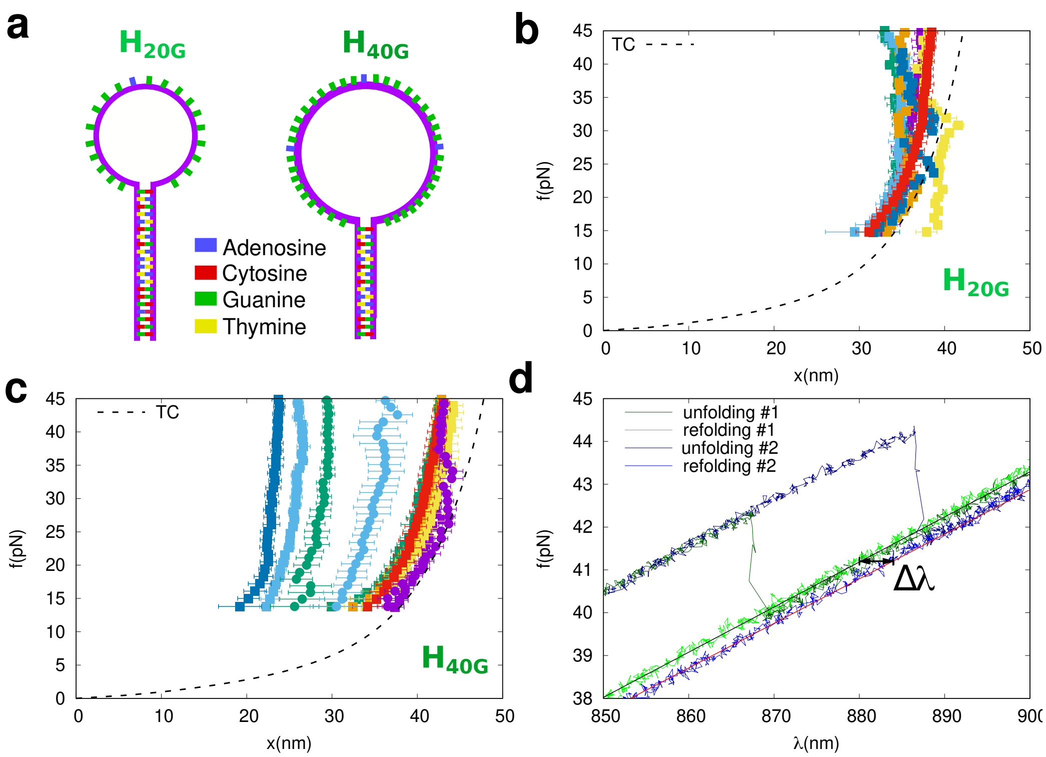

Figure S14: H_{20G} **and** H_{40G} **elasticity. a.** Schematic depictions of the H_{20G} and H_{40G} hairpins. **b.** Average force-extension curves (FECs) for 6 different molecules of the molecule with 20 dG in the loop. The dashed lines indicate the ideal elasticity modeled using the thick chain model that should be recovered at high forces. **c.** Average force-extension curves (FECs) for 9 different molecules of the molecule with 40 dG in the loop. The dashed lines indicate the ideal elasticity modeled using the thick chain model that should be recovered at high forces. **d.** Zoom of consecutive unfolding and refolding FDC trajectories. The released extension upon unfolding varies from one cycle to another, suggesting the formation of G-quadruplex structures. The error bars are statistical errors associated to the average between different the different cycles obtained for each molecule.

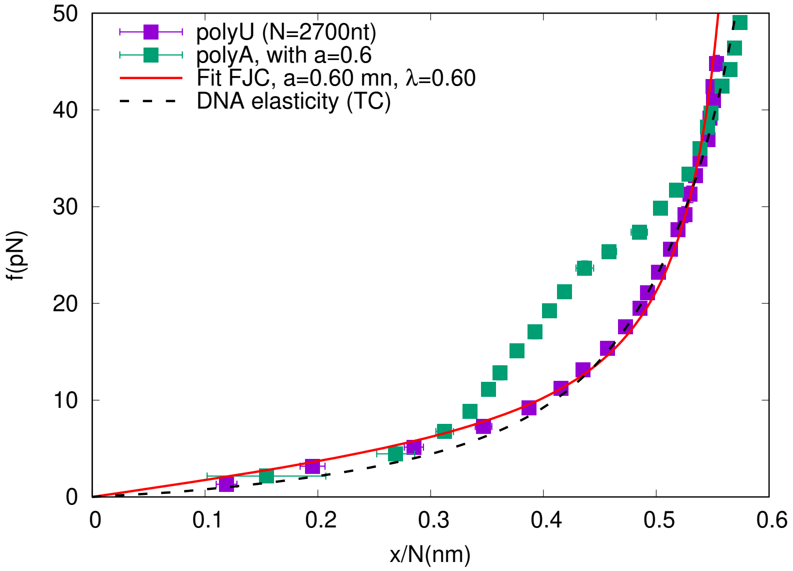

Figure S15: **Characterization of the unstacked elasticity for RNA.** Scaled FEC for poly-U (magenta, squares) and poly-rA molecules (green, squares), data from Ref.[15]. The FJC successfully fits the poly-U data (red curve) with a contour length per base of $a = 0.60$nm and a Kuhn's length of $\lambda = 0.60$nm. The black dashed line shows the TC model used to reproduce the FECs for the ssDNA released for H_0 (Main Sec. Unstacked elasticity).

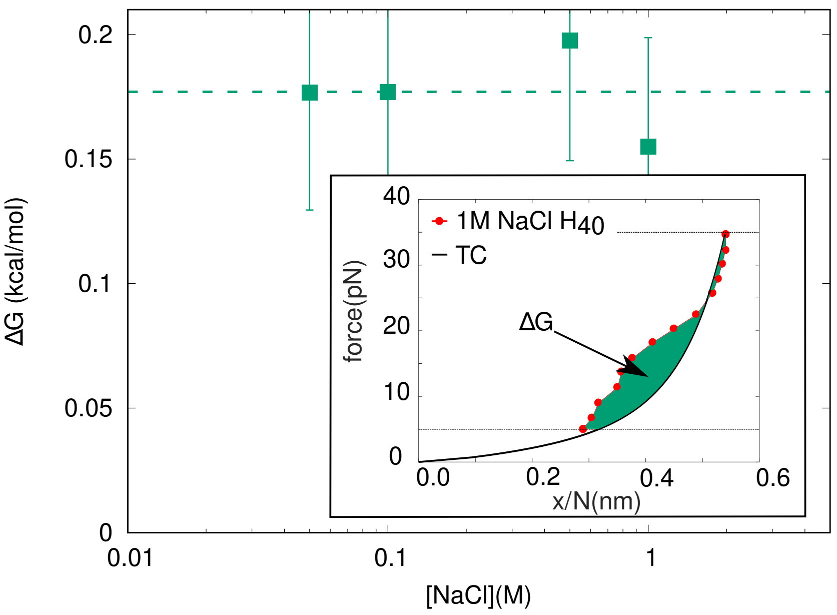

Figure S16: **Estimation of the free-energy of stacking from the experimental FECs.** The area between the experimental points and the unstacked elasticity is used to estimate the free energy difference due to stacking, ΔG. The main plot shows ΔG as a function of the salt concentration (in logarithmic scale). The inset shows a schematic representation of how the area is calculated: $\Delta G = \int_{f_{min}}^{f_{max}} x_U(f')df' - \int_{f_{min}}^{f_{max}} x(f')df'$, with $x(f)$ being the experimental points (in red) and $x_U(f)$ the unstacked elasticity described using the TC model (See main text Sec. Unstacked Elasticity). The ΔG estimation obtained with this method can not be directly compared to previous measurements of stacking free energy [10], because the low-force reference state is different. In previous studies, the reference state is at zero force, whereas here the low-force reference state is at about 5 pN.

Table S1: Oligonucleotides (from *Merck-Sigma Aldrich*) used for the synthesis of the DNA constructs. The loop region is shown in bold while the handle regions are shown in italics.

**Name**	**Sequence**
Sequence oligonucleotide for H_0	5′-Biotin-*AGT TAG TGG TGG AAA CAC AGT GCC AGC GC* GCG AGC CAT AAT CTC ATC TG **GTT TTT TTT TTT TTT TTT TT** CAG ATG AGA TTA TGG CTC GC *GAC TTC ACT AAT ACG ACT CAC TAT AGG GA*-3′
Sequence oligonucleotide for H_{20}	5′-Biotin-*AGT TAG TGG TGG AAA CAC AGT GCC AGC GC* GCG AGC CAT AAT CTC ATC TG **GAA AAA AAA AAA AAA AAA AA** CAG ATG AGA TTA TGG CTC GC *GAC TTC ACT AAT ACG ACT CAC TAT AGG GA*-3′
Sequence oligonucleotide for H_{40}	5′-Biotin-*AGT TAG TGG TGG AAA CAC AGT GCC AGC GC* GCC ATA TCT CAT CTG **GAA AAA AAA AAA AAA AAA AAA AAA AAA AAA AAA AAA AAA A** CAG ATG AGA TAT GGC *GAC TTC ACT AAT ACG ACT CAC TAT AGG GA*-3′
Sequence oligonucleotide for L_{40}	5′-Biotin-*AGT TAG TGG TGG AAA CAC AGT GCC AGC GC* **AAA AAA AAA AAA AAA AAA AAA AAA AAA AAA AAA AAA AAA A** *GAC TTC ACT AAT ACG ACT CAC TAT AGG GA*-3′
Sequence oligonucleotide for H_{20GA}	5′-Biotin-*AGT TAG TGG TGG AAA CAC AGT GCC AGC GC* GCG AGC CAT AAT CTC ATC TG **GAG AGA GAG AGA GAG AGA GA** CAG ATG AGA TTA TGG CTC GC *GAC TTC ACT AAT ACG ACT CAC TAT AGG GA*-3′
Sequence oligonucleotide for H_{40GA}	5′-Biotin-*AGT TAG TGG TGG AAA CAC AGT GCC AGC GC* GCC ATA TCT CAT CTG **GAG AGA GAG AGA GAG AGA GAG AGA GAG AGA GAG AGA GAG A** CAG ATG AGA TAT GGC *GAC TTC ACT AAT ACG ACT CAC TAT AGG GA*-3′
Sequence oligonucleotide for H_{20G}	5′-Biotin-*AGT TAG TGG TGG AAA CAC AGT GCC AGC GC* GCG AGC CAT AAT CTC ATC TG **GGG GGG GGG AGG GGG GGG GG** CAG ATG AGA TTA TGG CTC GC *GAC TTC ACT AAT ACG ACT CAC TAT AGG GA*-3′
Sequence oligonucleotide for H_{40G}	5′-Biotin-*AGT TAG TGG TGG AAA CAC AGT GCC AGC GC* GCC ATA TCT CAT CTG **GGG GGG GGG AGG GGG GGG GAG GGG GGG GGA GGG GGG GGG G** CAG ATG AGA TAT GGC *GAC TTC ACT AAT ACG ACT CAC TAT AGG GA*-3′
Blocking-splint	5′-*CGT ATT AGT GAA GTC* TTTT *GCG CTG GCA CTG TGT TTC CAC CAC TAA CT-3′*
Complementary handle B	5′ -*TCC CTA TAG TGA GT*-3′

Table S2: **Fixed a at all salt conditions.** TC model best-fitting parameters obtained by simultaneously fitting all salt-conditions and sequences, imposing a fixed a independently of the salt and sequence. The fit has a $\chi^2 = 147$, $\chi^2_\nu = 1.33$, $AIC = 65.9$ and leads to $a = 1.34 \pm 0.11$ nm.

Molecule	NaCl concentration(mM)	l_U(nm)	Δ(nm)
H_0	10	0.614(13)	0.88(7)
7kb from Ref.[7]	10(+glyoxal)	0.600(5)	0.92(13)
H_0	50	0.596(7)	0.79(8)
H_0	100	0.590(6)	0.81(10)
H_0	500	0.594(7)	0.61(8)
H_0	1000	0.582(7)	0.71(9)
7kb from Ref.[7]	1000(+glyoxal)	0.580(8)	0.92(8)

Table S3: **Fixed l_U at all salt conditions.** TC model best-fitting parameters obtained by simultaneously fitting all salt-conditions and sequences, imposing a fixed l_U independently of the salt and sequence. The fit has a $\chi^2 = 36.1$, $\chi^2_\nu = 0.33$, $AIC = -109$ and leads to $l_U = 0.591 \pm 0.007$ nm.

Molecule	NaCl concentration(mM)	Δ(nm)	a(nm)
H_0	10	1.08(14)	1.83(7)
7kb from Ref.[7]	10(+glyoxal)	1.01(14)	1.53(8)
H_0	50	0.82(11)	1.43(5)
H_0	100	0.80(12)	1.33(6)
H_0	500	0.61(7)	1.36(5)
H_0	1000	0.67(7)	1.22(5)
7kb from Ref.[7]	1000(+glyoxal)	0.64(6)	1.21(6)

Table S4: **Fixed Δ at all salt conditions.** TC model best-fitting parameters obtained by simultaneously fitting all salt-conditions and sequences, imposing a fixed Δ independently of the salt and sequence. The fit has a $\chi^2 = 17420$, $\chi^2_\nu = 158$, $AIC = 663$ and leads to $\Delta = 0.61 \pm 0.11$ nm.

Molecule	NaCl concentration(mM)	l_0(nm)	a(nm)
H_0	10	0.59(2)	1.9(8)
7kb from Ref.[7]	10(+glyoxal)	0.59(2)	1.5(4)
H_0	50	0.571(13)	1.8(2)
H_0	100	0.621(11)	0.96(17)
H_0	500	0.611(10)	1.13(10)
H_0	1000	0.605(11)	1.08(10)
7kb from Ref.[7]	1000(+glyoxal)	0.605(15)	1.06(14)

Table S5: **Fixed a and l_U at all salt conditions.** TC model best-fitting parameters obtained by simultaneously fitting all salt-conditions and sequences, imposing a fixed a and l_U independently of the salt and sequence. The fit has a $\chi^2 = 3.09$ nm^2, $\chi^2_\nu = 0.266$ nm^2, $AIC = -147$ and leads to $a = 0.81 \pm 0.14$ nm and $l_U = 0.641 \pm 0.013$ nm.

Molecule	NaCl concentration(mM)	Δ(nm)
H_0	10	0.58(9)
7kb from Ref.[7]	10(+glyoxal)	0.63(9)
H_0	50	0.54(8)
H_0	100	0.52(8)
H_0	500	0.50(7)
H_0	1000	0.48(7)
7kb from Ref.[7]	1000(+glyoxal)	0.48(8)

Table S6: **Standard deviation and errors of the extension** Results are shown for the values of a single molecule (averaging over N_{cycles} obtained cycles) and the averages obtained from averaging 4-7 different molecules. The values here provided are taken at $\sim$ 20pN but are similar throughout the force-range studied (Figs. S3, S5, S8).

Molecule and salt condition	σ_{cycles} (nm)	N_{cycles}	$err._{cycles}$/nt (nm)	σ_{mol} (nm)	N_{mol}	$err._{mol.}$/nt (nm)
H_0, 10 mM NaCl	1.6	22	0.005	1.8	6	0.010
H_0, 50 mM NaCl	1.3	11	0.005	0.9	5	0.005
H_0, 100 mM NaCl	2.1	12	0.008	1.6	5	0.010
H_0, 500 mM NaCl	2.1	23	0.006	0.5	5	0.003
H_0, 1000 mM NaCl	1.5	7	0.008	1.3	4	0.009
H_0, 10 mM $MgCl_2$	2.7	21	0.008	1.4	7	0.007
H_{20}, 10 mM $MgCl_2$	1.8	86	0.003	1.1	6	0.006
H_{40}, 10 mM $MgCl_2$	2.2	12	0.007	0.9	5	0.005
L_{40}, 10 mM $MgCl_2$	1.8	7	0.007	1.3	4	0.008
H_{40}, 50 mM NaCl	1.4	13	0.005	0.7	4	0.005
H_{40}, 100 mM NaCl	1.5	21	0.004	1.1	5	0.006
H_{40}, 500 mM NaCl	1.6	70	0.002	1.7	5	0.010
H_{40}, 1000 mM NaCl	2.1	56	0.003	0.7	4	0.005
H_{20AG}, 10 mM $MgCl_2$	1.4	55	0.003	0.7	7	0.004
H_{40AG}, 10 mM $MgCl_2$	1.3	52	0.002	0.8	4	0.005

References

[1] Steven B. Smith, Yujia Cui, and Carlos Bustamante. Overstretching B-DNA: The elastic response of individual double-stranded and single-stranded DNA molecules. Science, 271(5250):795–799, 1996.

[2] John F Marko and Eric D Siggia. Stretching DNA. Macromolecules, 28(26):8759–8770, 1995.

[3] C Bouchiat, MD Wang, J-F Allemand, T Strick, SM Block, and V Croquette. Estimating the persistence length of a worm-like chain molecule from force-extension measurements. Biophysical journal, 76(1):409–413, 1999.

[4] O. A. Saleh, D. B. McIntosh, P. Pincus, and N. Ribeck. Nonlinear low-force elasticity of single-stranded DNA molecules. Phys. Rev. Lett., 102:068301, Feb 2009.

[5] David R. Jacobson, Dustin B. McIntosh, Mark J. Stevens, Michael Rubinstein, and Omar A. Saleh. Single-stranded nucleic acid elasticity arises from internal electrostatic tension. Proceedings of the National Academy of Sciences, 114(20):5095–5100, 2017.

[6] Ngo Minh Toan, Davide Marenduzzo, and Cristian Micheletti. Inferring the diameter of a biopolymer from its stretching response. Biophysical Journal, 89(1):80–86, 2005.

[7] X. Viader-Godoy, C. R. Pulido, B. Ibarra, M. Manosas, and F. Ritort. Cooperativity-dependent folding of single-stranded DNA. Phys. Rev. X, 11:031037, Aug 2021.

[8] Nuria Forns, Sara de Lorenzo, Maria Manosas, Kumiko Hayashi, Josep Maria Huguet, and Felix Ritort. Improving signal/noise resolution in single-molecule experiments using molecular constructs with short handles. Biophysical Journal, 100(7):1765–1774, 2011.

[9] Jay R. Wenner, Mark C. Williams, Ioulia Rouzina, and Victor A. Bloomfield. Salt dependence of the elasticity and overstretching transition of single DNA molecules. Biophysical Journal, 82(6):3160–3169, 2002.

[10] DustinB. McIntosh, Gina Duggan, Quentin Gouil, and OmarA. Saleh. Sequence-dependent elasticity and electrostatics of single-stranded DNA: Signatures of base-stacking. Biophysical Journal, 106(3):659 – 666, 2014.

[11] Roland R. Netz. Strongly stretched semiflexible extensible polyelectrolytes and DNA. Macromolecules, 34(21):7522–7529, Oct 2001.

[12] Gerald S. Manning. The persistence length of dna is reached from the persistence length of its null isomer through an internal electrostatic stretching force. Biophysical Journal, 91(10):3607–3616, 2006.

[13] D Marenduzzo, C Micheletti, H Seyed-allaei, A Trovato, and A Maritan. Continuum model for polymers with finite thickness. Journal of Physics A: Mathematical and General, 38(17):L277, apr 2005.

[14] Alessandro Bosco, Joan Camunas-Soler, and Felix Ritort. Elastic properties and secondary structure formation of single-stranded DNA at monovalent and divalent salt conditions. Nucleic acids research, 42(3):2064–2074, 2014.

[15] Yeonee Seol, Gary M. Skinner, Koen Visscher, ARNAud Buhot, and Avraham Halperin. Stretching of homopolymeric RNA reveals single-stranded helices and base-stacking. Phys. Rev. Lett., 98:158103, Apr 2007.
